# Supplementary material for: An Efficient Metal-Free Oxidative Esterification and Amination of Benzyl C–H Bond
Source: Molecules. 2020 Mar 27;25(7):1527. doi: 10.3390/molecules25071527 (PMC7180972; doi:10.3390/molecules25071527)
Supplement: Supplementary file 1 [file molecules-25-01527-s001.pdf]

Supporting information for

An Efficient Metal-Free Oxidative Esterification and Amination of Benzyl C-H Bond

Saiwen Liu [1,*], Ru Chen [2], Guowen He [1] and Jin Zhang [1,*]

[1] College of Materials and Chemical Engineering, Hunan City University, Yiyang, 413000, China
[2] Yiyang Agriculture Products Quality Detect Center, Yiyang, Hunan 413000, China

* Correspondence: liusaiwen7@163.com

Table of Contents

Copies of ^{1}H and ^{13}C NMR spectra of products **S2-S30**

^{1}H and ^{13}C NMR spectra

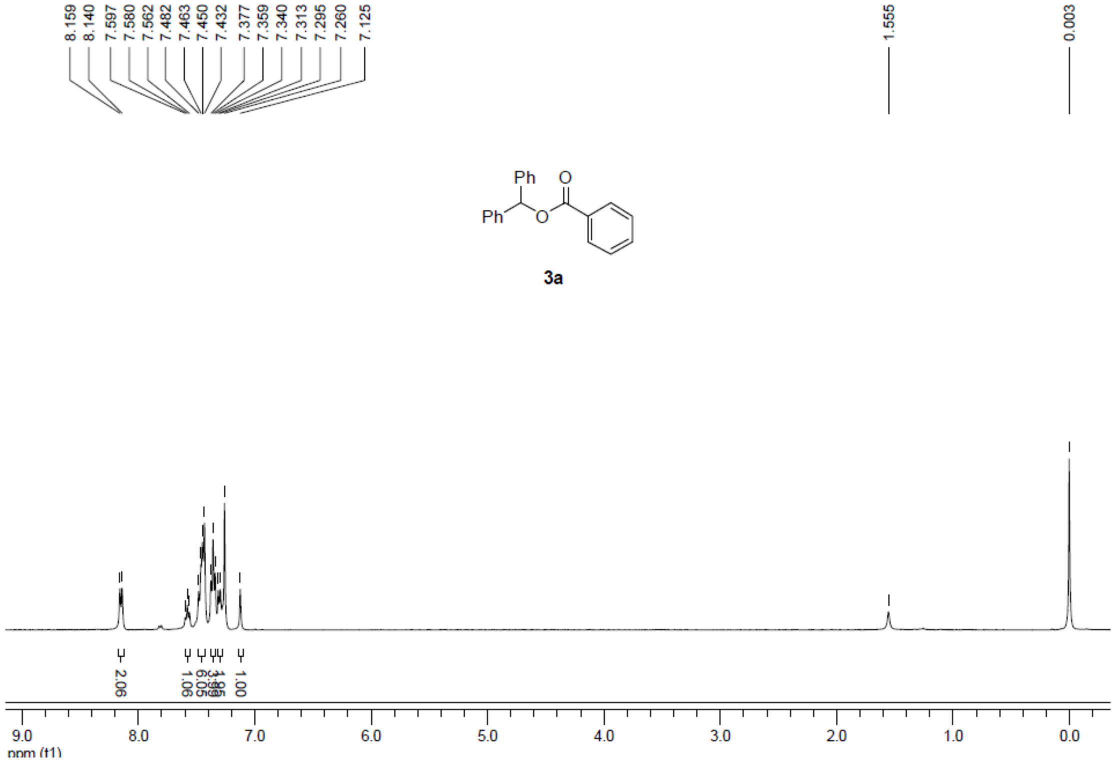

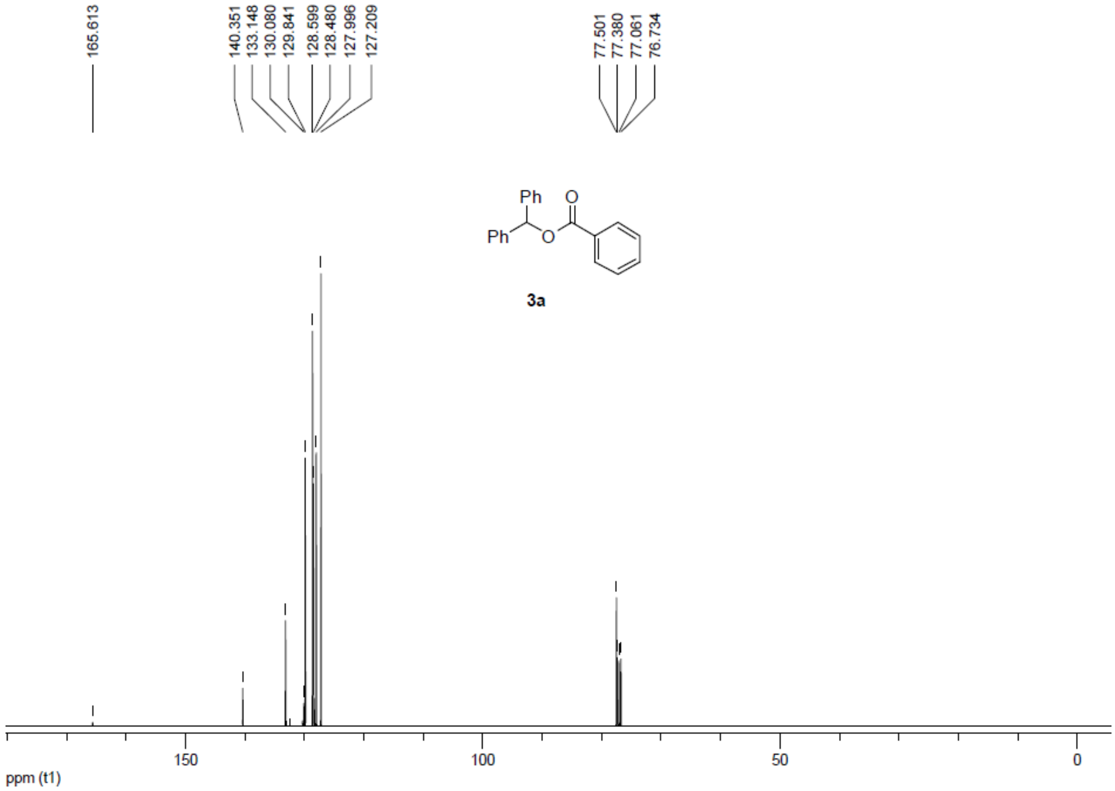

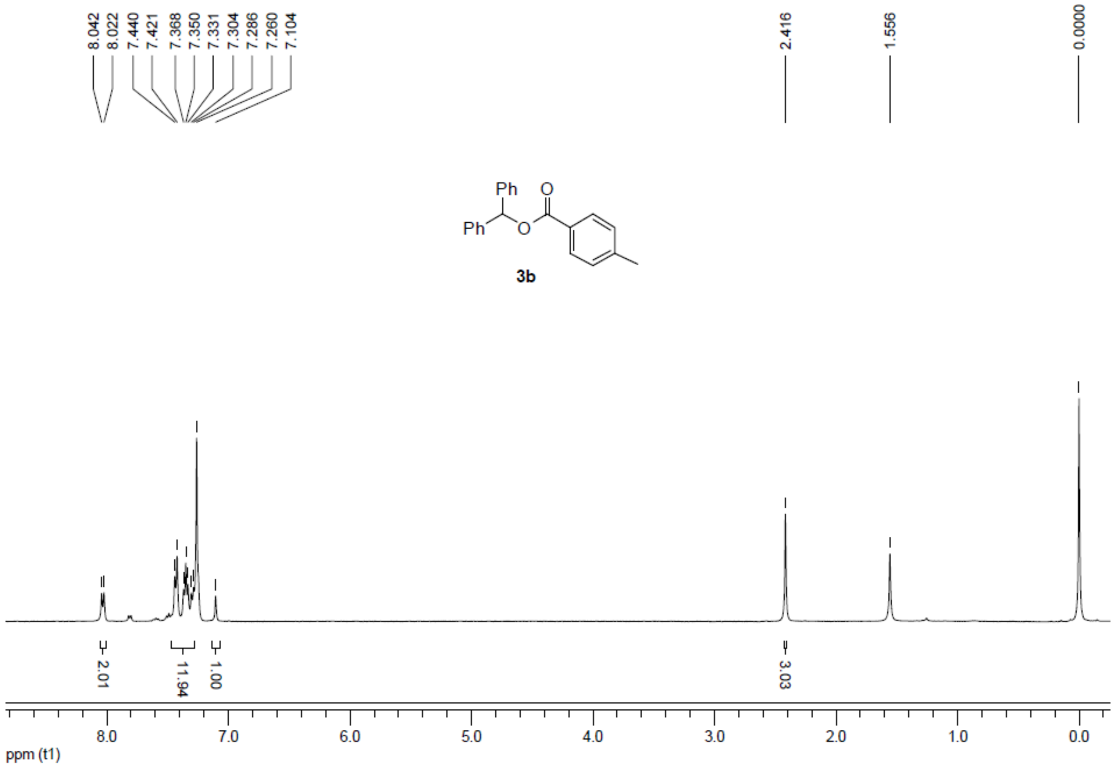

8.042
8.022
7.440
7.421
7.368
7.350
7.331
7.304
7.286
7.260
7.104
2.416
1.556
0.0000
Ph
O
Ph
O
3b
2.01
11.94
1.00
3.03
8.0
7.0
6.0
5.0
4.0
3.0
2.0
1.0
0.0
ppm (t1)

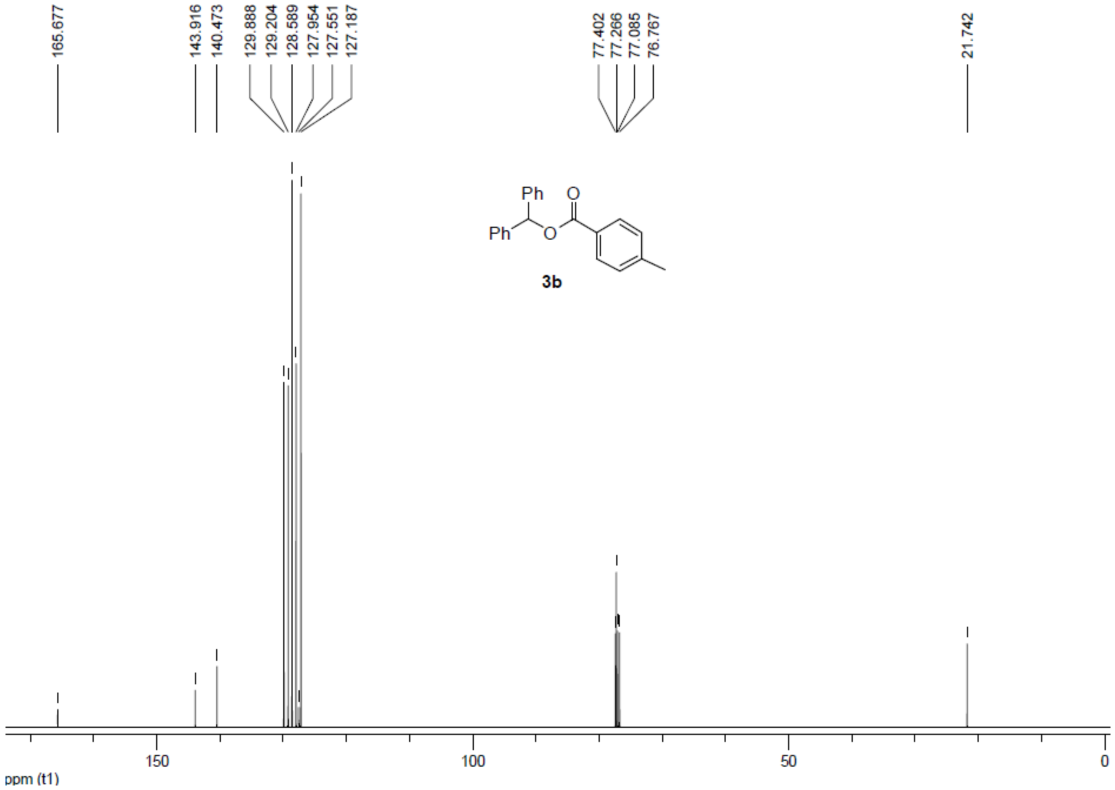

165.677
143.916
140.473
129.888
129.204
128.589
127.954
127.551
127.187
77.402
77.266
77.085
76.767
21.742
Ph
O
Ph
O
3b
150
100
50
0
ppm (t1)

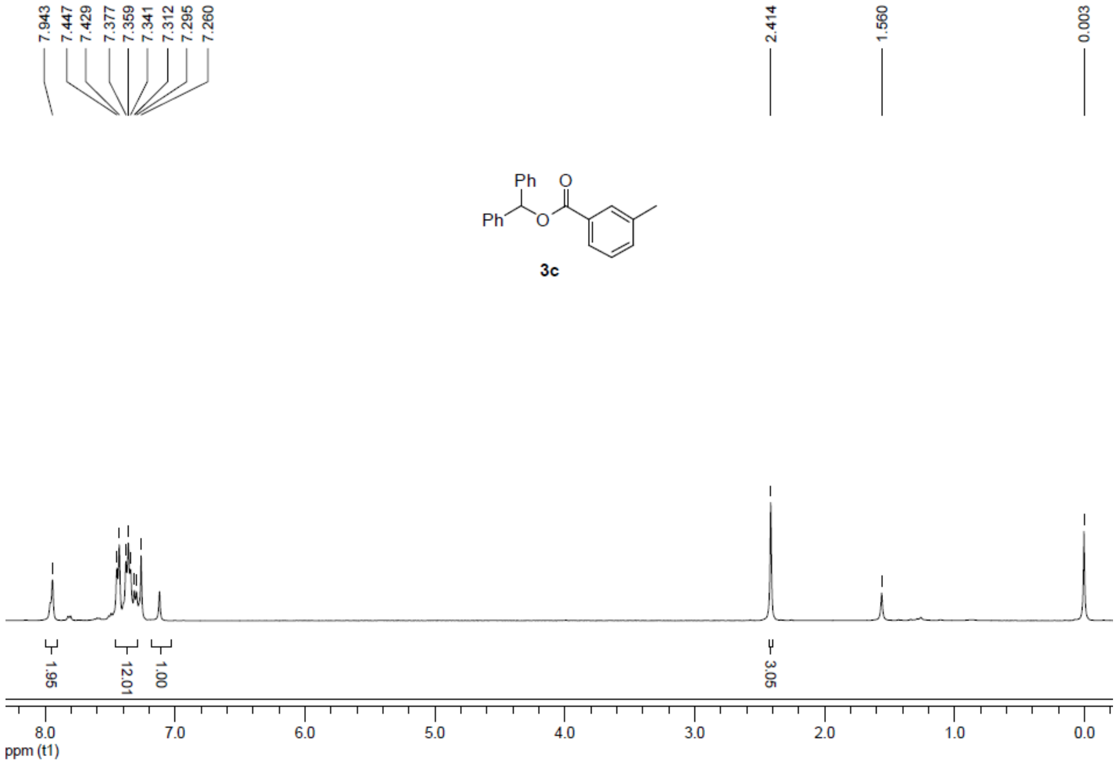
7.943
7.447
7.429
7.377
7.359
7.341
7.312
7.295
7.260
2.414
1.560
0.003
Ph
O
Ph
O
3c
1.95
12.01
1.00
3.05
8.0
7.0
6.0
5.0
4.0
3.0
2.0
1.0
0.0
ppm (t1)

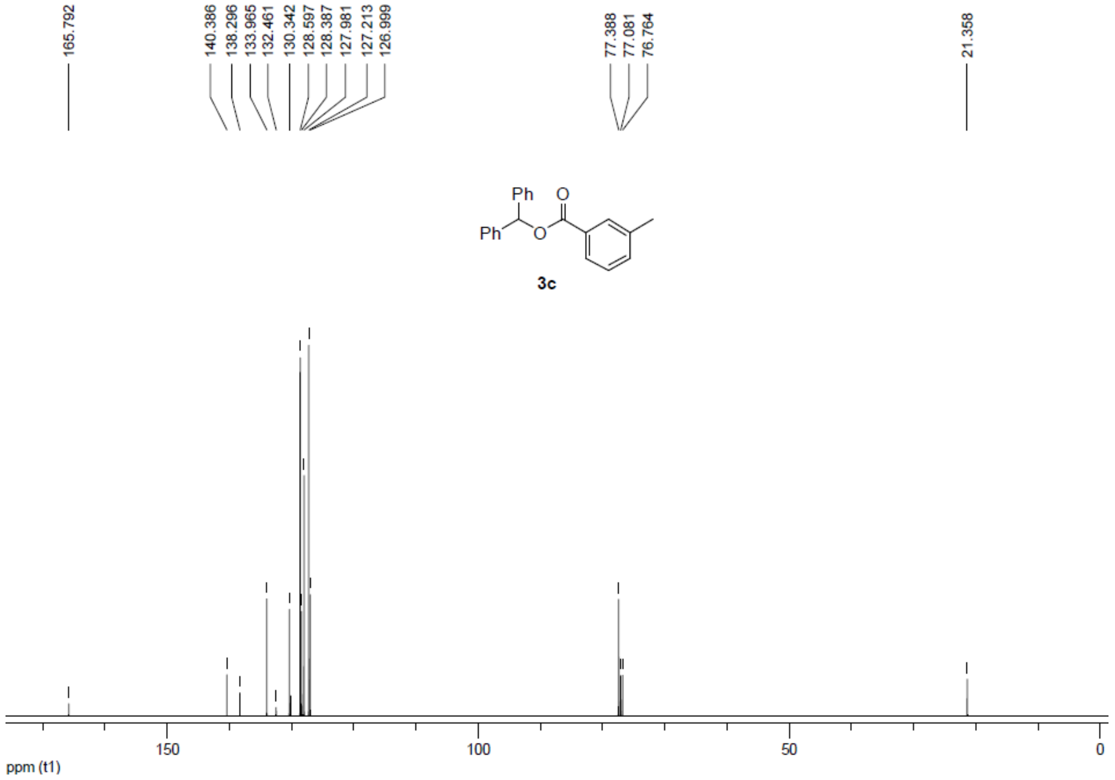
165.792
140.386
138.296
133.965
132.461
130.342
128.597
128.387
127.981
127.213
126.999
77.388
77.081
76.764
21.358
Ph
O
Ph
O
3c
150
100
50
0
ppm (t1)

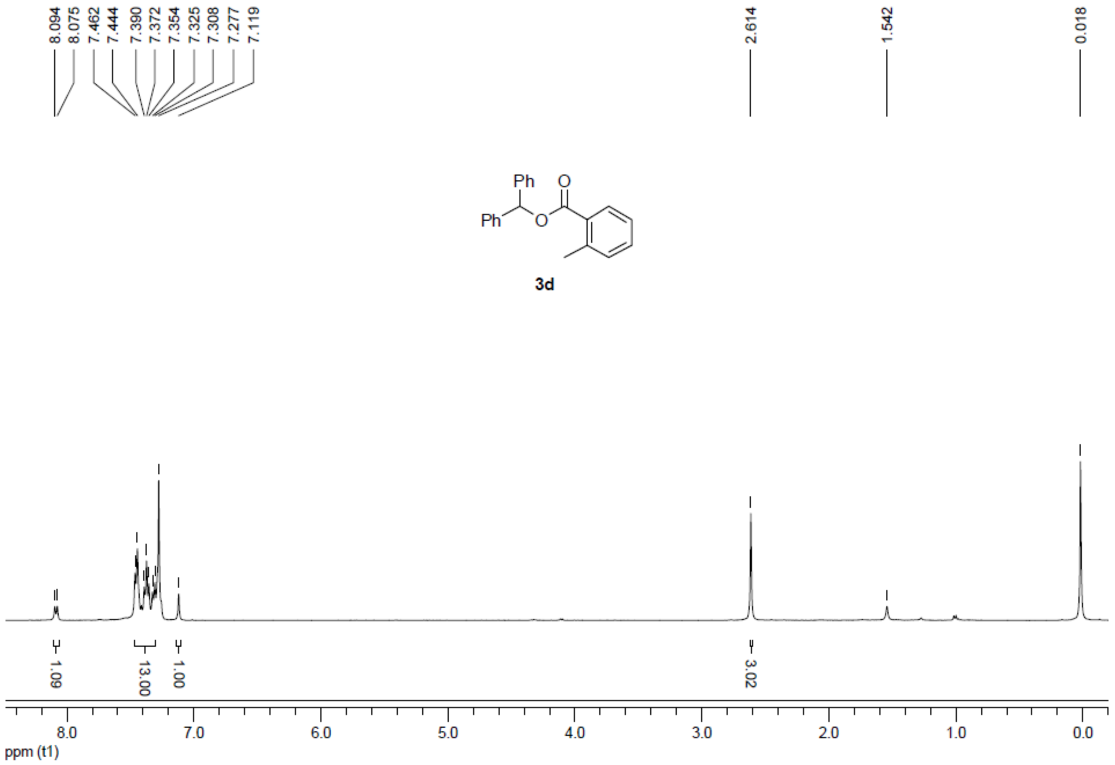
8.094
8.075
7.462
7.444
7.390
7.372
7.354
7.325
7.308
7.277
7.119
2.614
1.542
0.018
Ph
O
Ph
O
3d
1.09
13.00
1.00
3.02
8.0
7.0
6.0
5.0
4.0
3.0
2.0
1.0
0.0
ppm (t1)

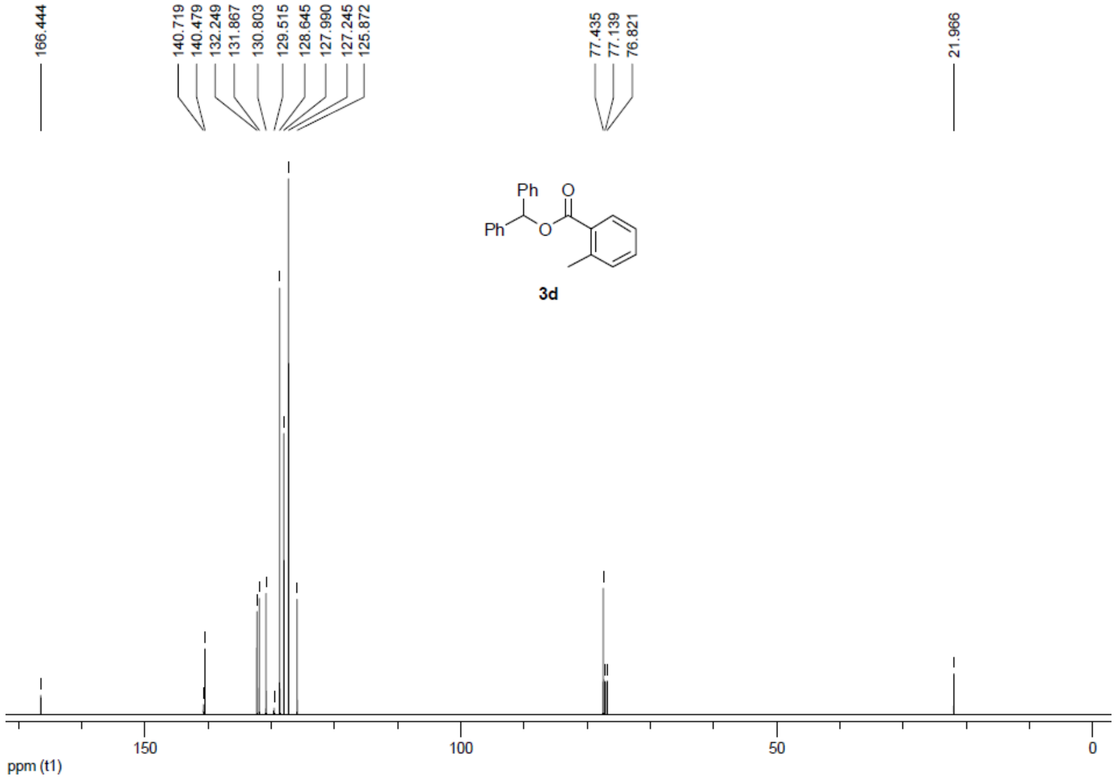
166.444
140.719
140.479
132.249
131.867
130.803
129.515
128.645
127.990
127.245
125.872
77.435
77.139
76.821
21.966
Ph
O
Ph
O
3d
150
100
50
0
ppm (t1)

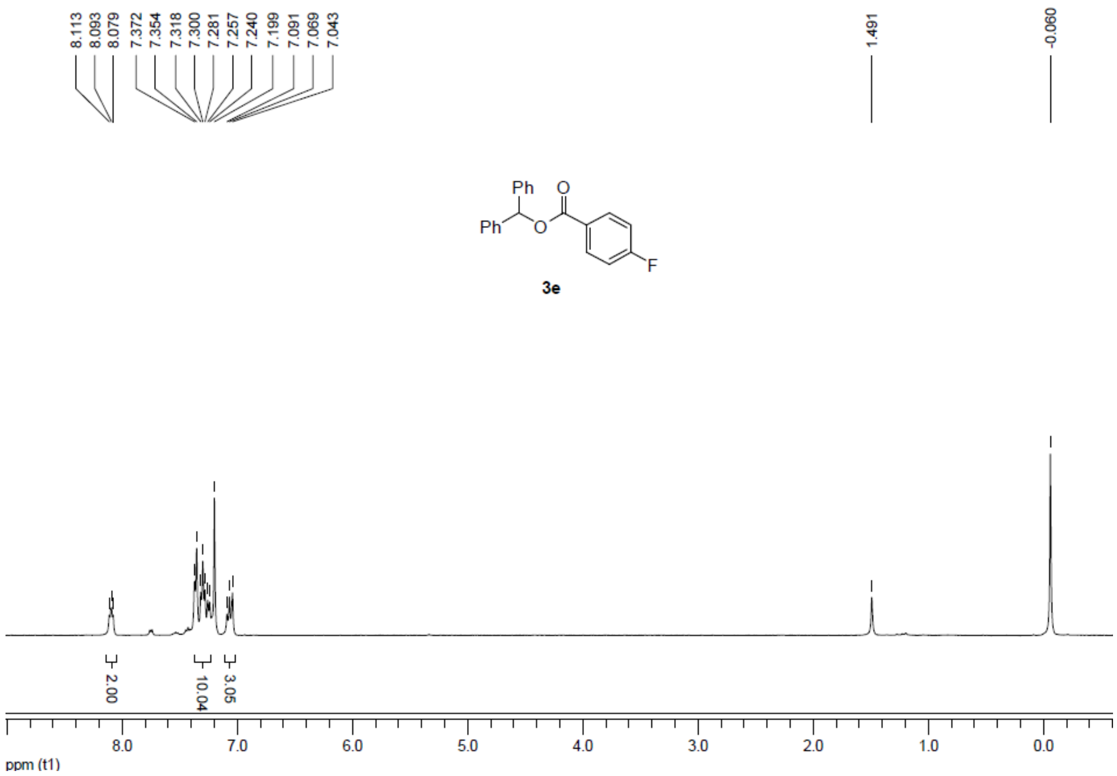
8.113
8.093
8.079
7.372
7.354
7.318
7.300
7.281
7.257
7.240
7.199
7.091
7.069
7.043
1.491
-0.060
Ph
O
Ph
O
F
3e
2.00
10.04
3.05
8.0
7.0
6.0
5.0
4.0
3.0
2.0
1.0
0.0
ppm (t1)

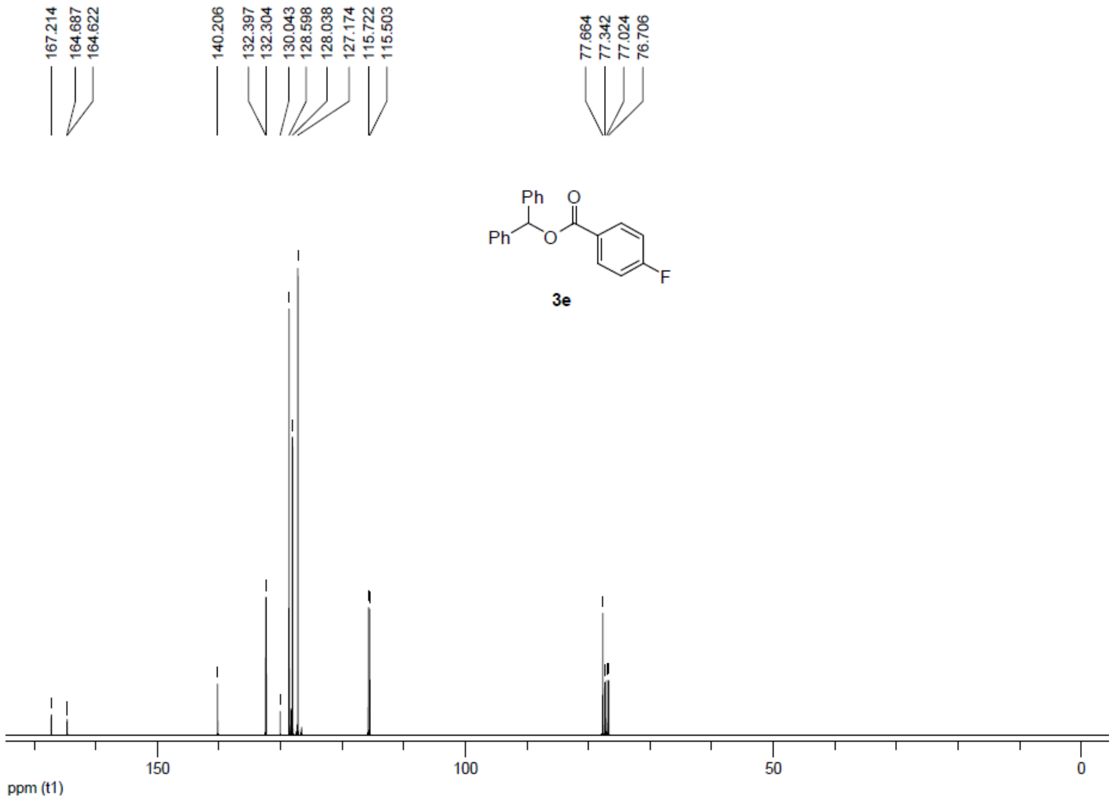
167.214
164.687
164.622
140.206
132.397
132.304
130.043
128.598
128.038
127.174
115.722
115.503
77.664
77.342
77.024
76.706
Ph
O
Ph
O
F
3e
150
100
50
0
ppm (t1)

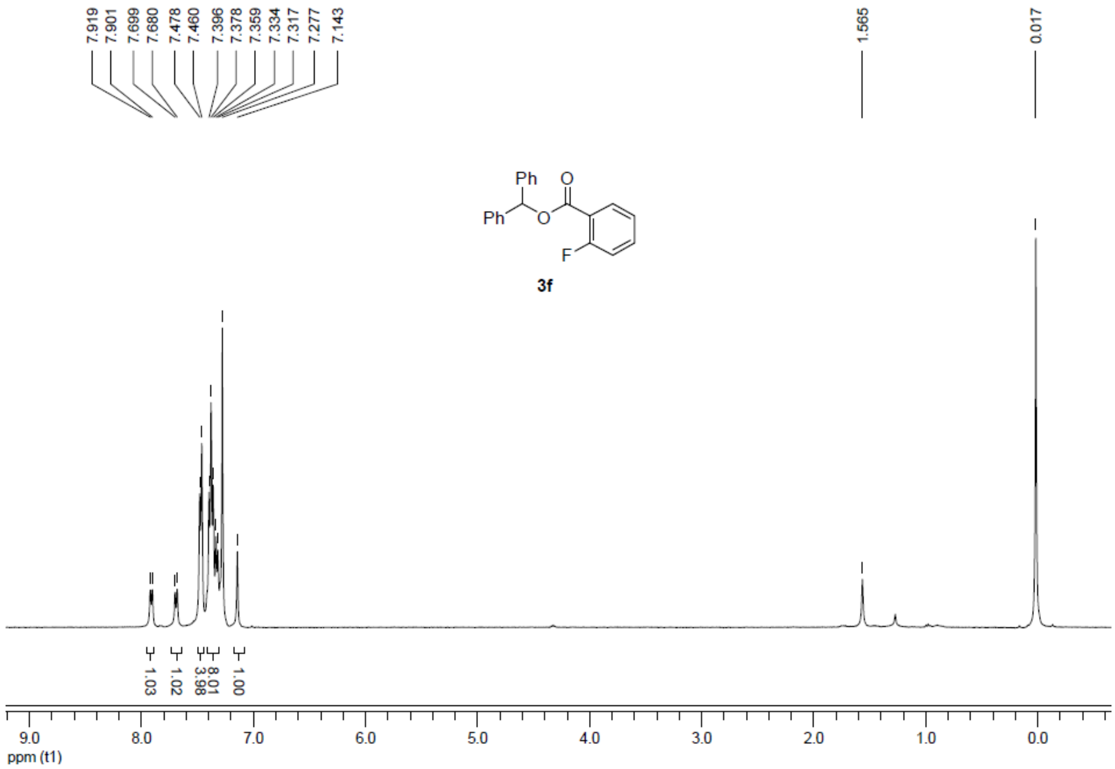

7.919
7.901
7.699
7.680
7.478
7.460
7.396
7.378
7.359
7.334
7.317
7.277
7.143
1.565
0.017
Ph
O
Ph
O
F
3f
1.03
1.02
3.98
8.01
1.00
9.0
8.0
7.0
6.0
5.0
4.0
3.0
2.0
1.0
0.0
ppm (t1)

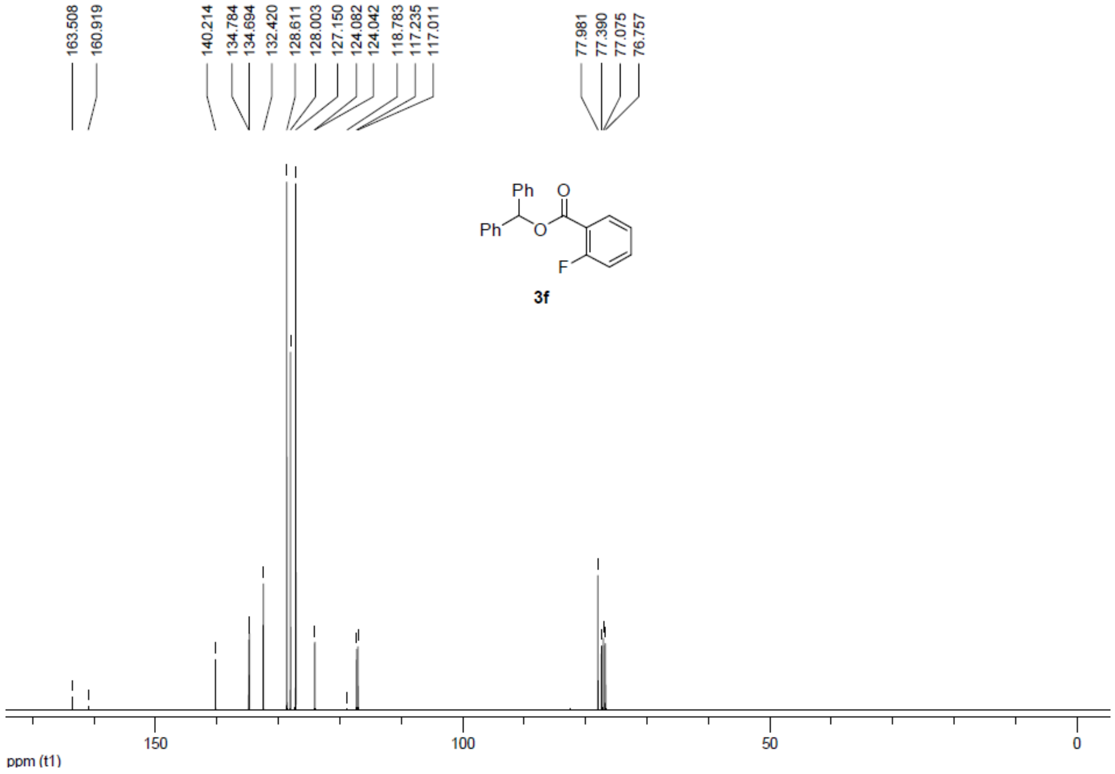

163.508
160.919
140.214
134.784
134.694
132.420
128.611
128.003
127.150
124.082
124.042
118.783
117.235
117.011
77.981
77.390
77.075
76.757
Ph
O
Ph
O
F
3f
150
100
50
0
ppm (t1)

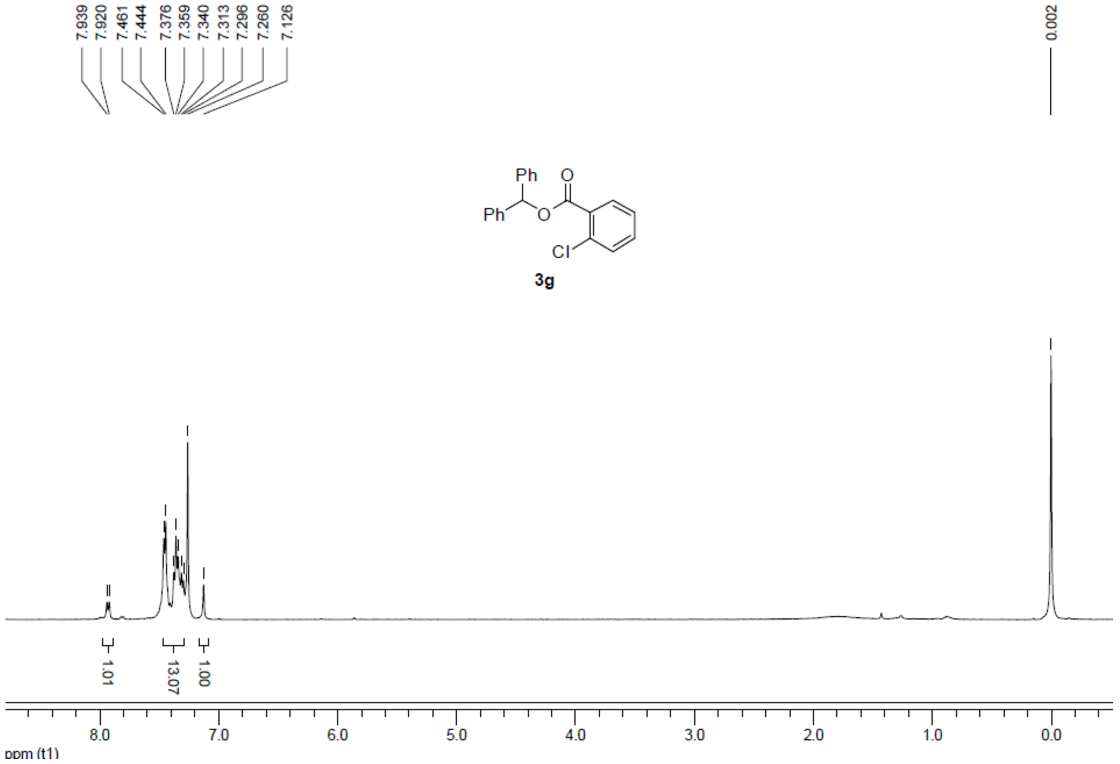
7.939
7.920
7.461
7.444
7.376
7.359
7.340
7.313
7.296
7.260
7.126
0.002
Ph
O
Ph
O
Cl
3g
1.01
13.07
1.00
8.0
7.0
6.0
5.0
4.0
3.0
2.0
1.0
0.0
ppm (t1)

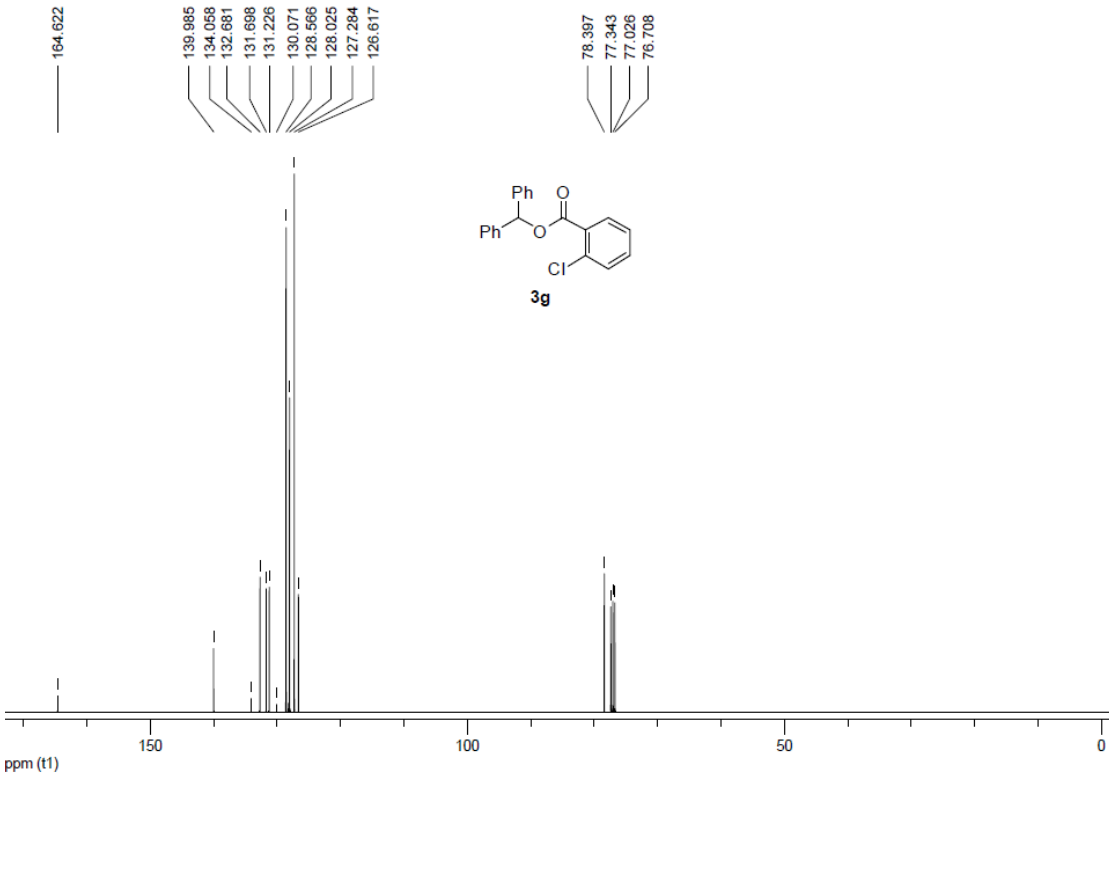
164.622
139.985
134.058
132.681
131.698
131.226
130.071
128.566
128.025
127.284
126.617
78.397
77.343
77.026
76.708
Ph
O
Ph
O
Cl
3g
150
100
50
0
ppm (t1)

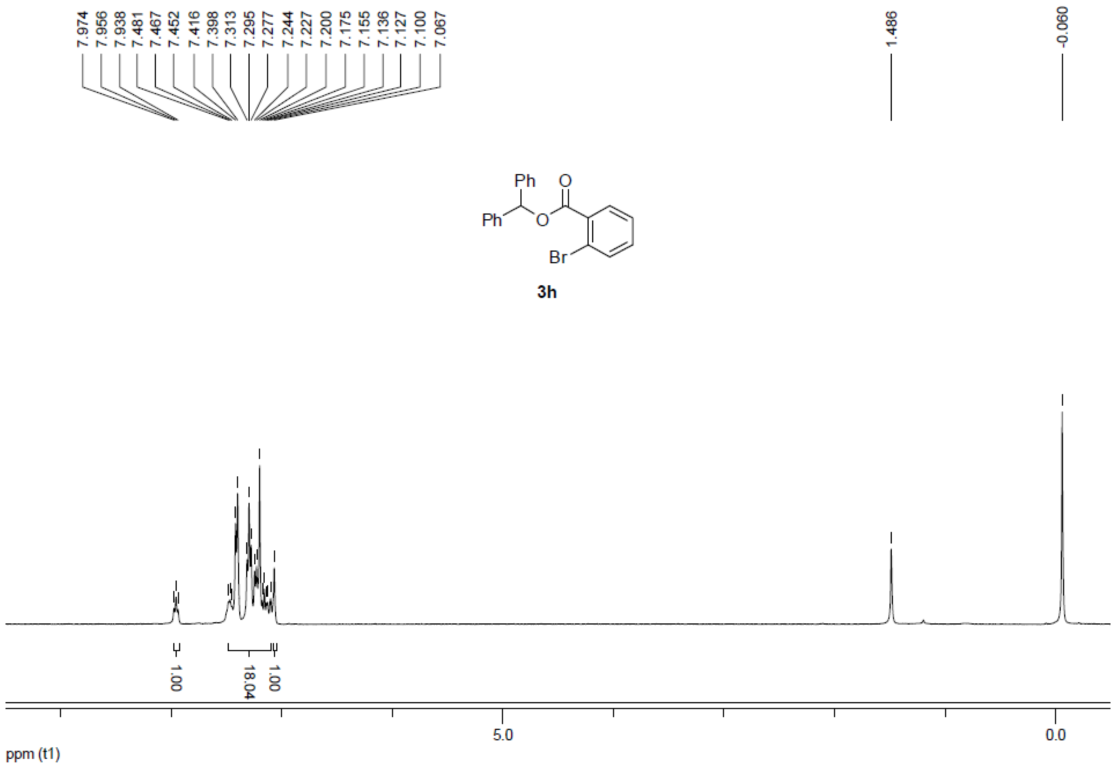

7.974
7.956
7.938
7.481
7.467
7.452
7.416
7.398
7.313
7.295
7.277
7.244
7.227
7.200
7.175
7.155
7.136
7.127
7.100
7.067
1.486
-0.060
Ph
O
Ph
O
Br
3h
1.00
18.04
1.00
5.0
0.0
ppm (t1)

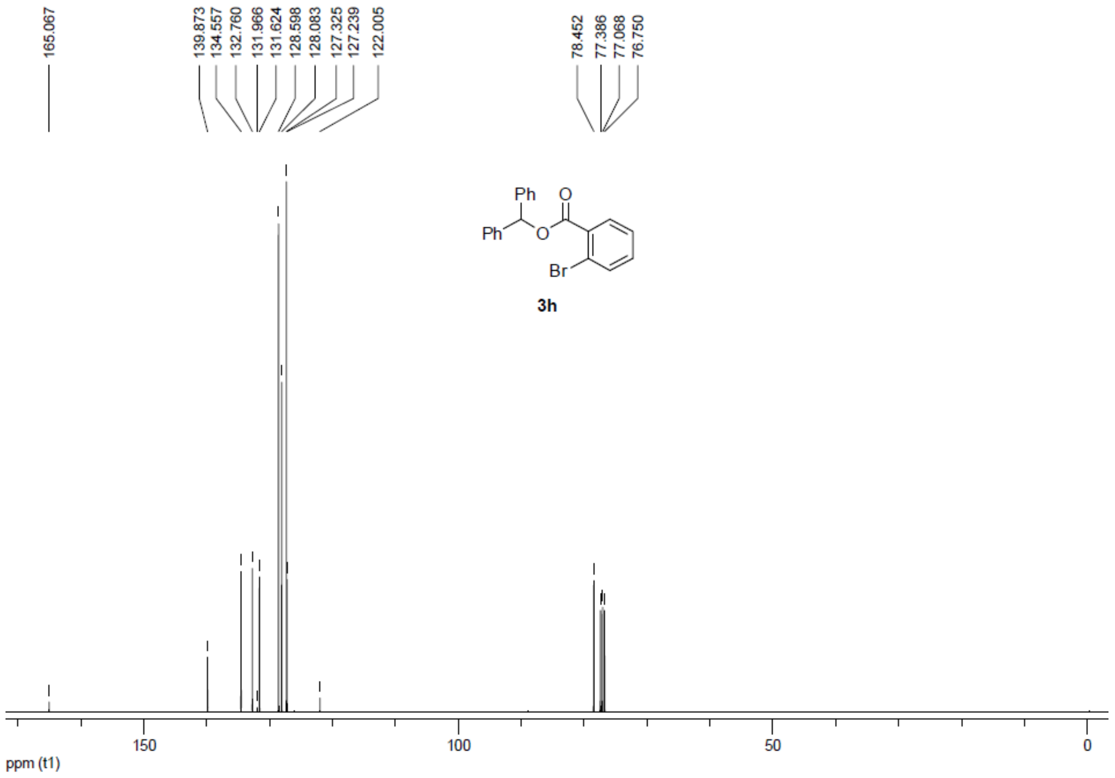

165.067
139.873
134.557
132.760
131.966
131.624
128.598
128.083
127.325
127.239
122.005
78.452
77.386
77.068
76.750
Ph
O
Ph
O
Br
3h
150
100
50
0
ppm (t1)

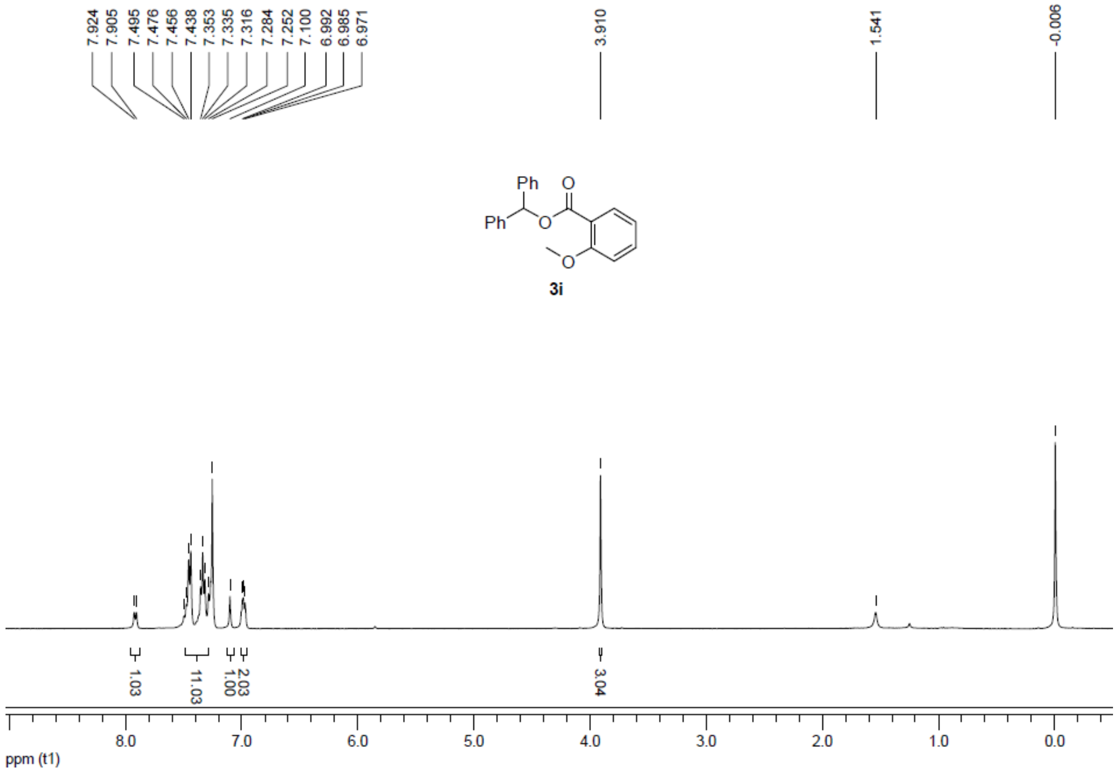

7.924
7.905
7.495
7.476
7.456
7.438
7.353
7.335
7.316
7.284
7.252
7.100
6.992
6.985
6.971
3.910
1.541
-0.006
Ph
O
Ph
O
O
3i
1.03
11.03
1.00
2.03
3.04
8.0
7.0
6.0
5.0
4.0
3.0
2.0
1.0
0.0
ppm (t1)

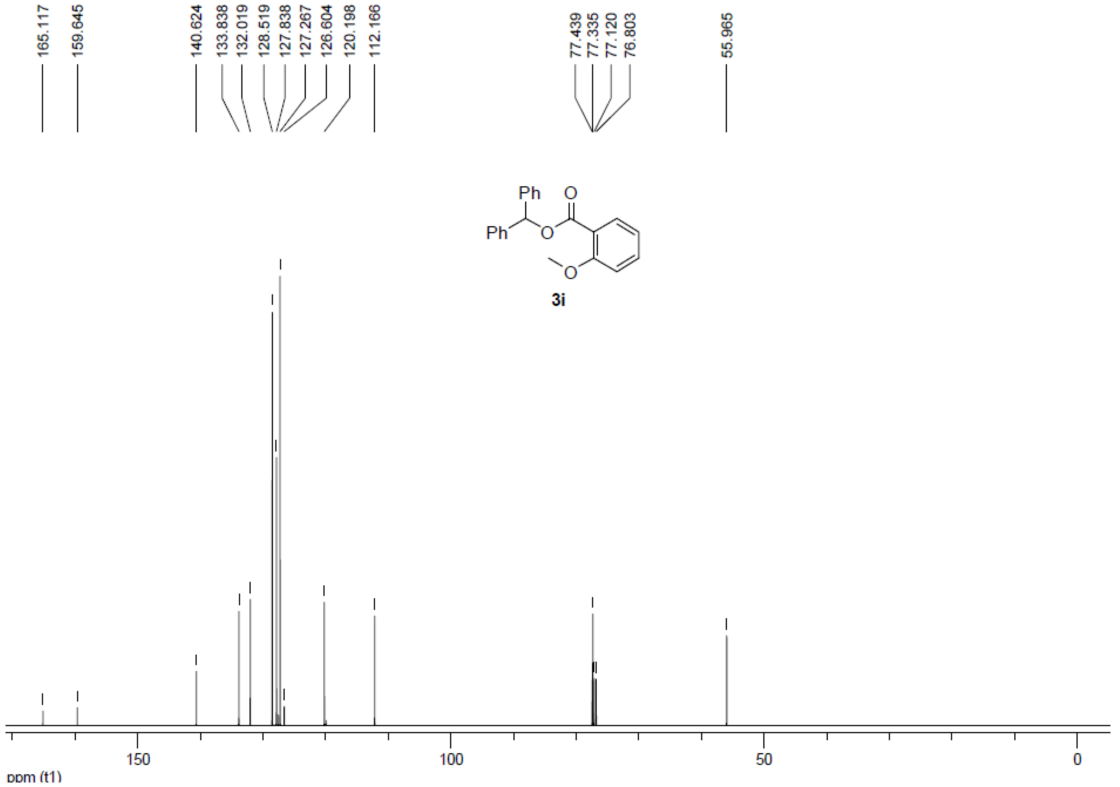

165.117
159.645
140.624
133.838
132.019
128.519
127.838
127.267
126.604
120.198
112.166
77.439
77.335
77.120
76.803
55.965
Ph
O
Ph
O
O
3i
150
100
50
0
ppm (t1)

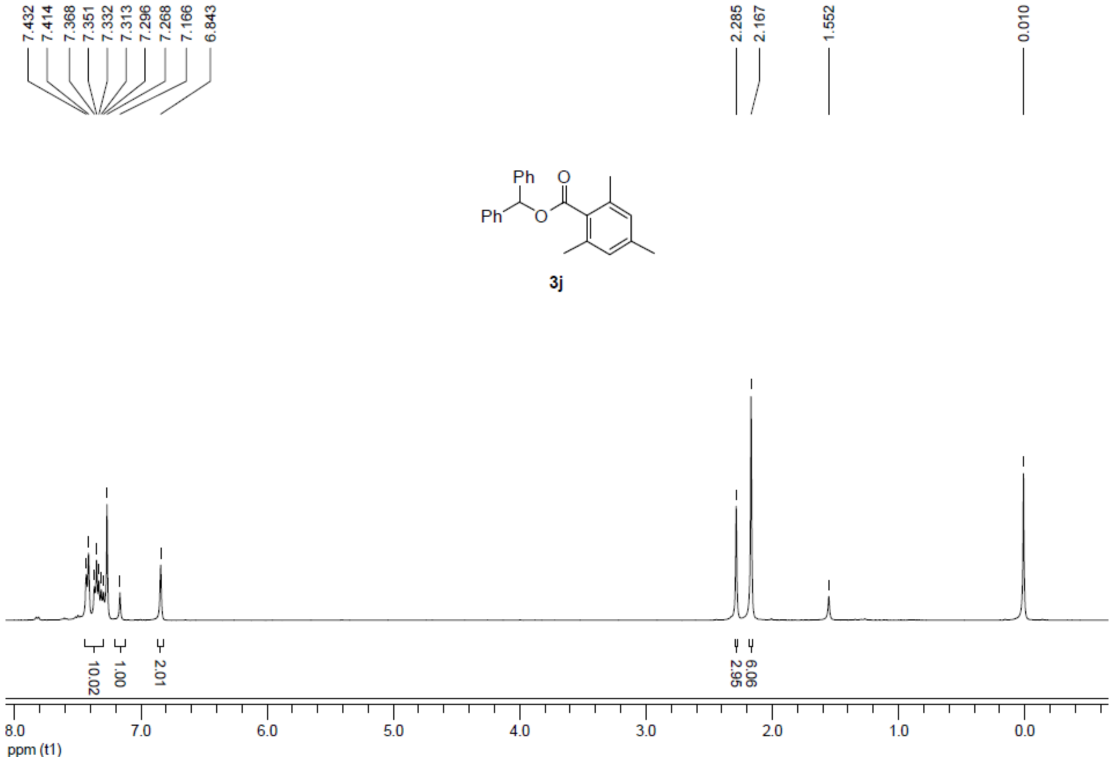
7.432
7.414
7.368
7.351
7.332
7.313
7.296
7.268
7.166
6.843
2.285
2.167
1.552
0.010
Ph
O
Ph
O
3j
10.02
1.00
2.01
2.95
6.06
8.0
7.0
6.0
5.0
4.0
3.0
2.0
1.0
0.0
ppm (t1)

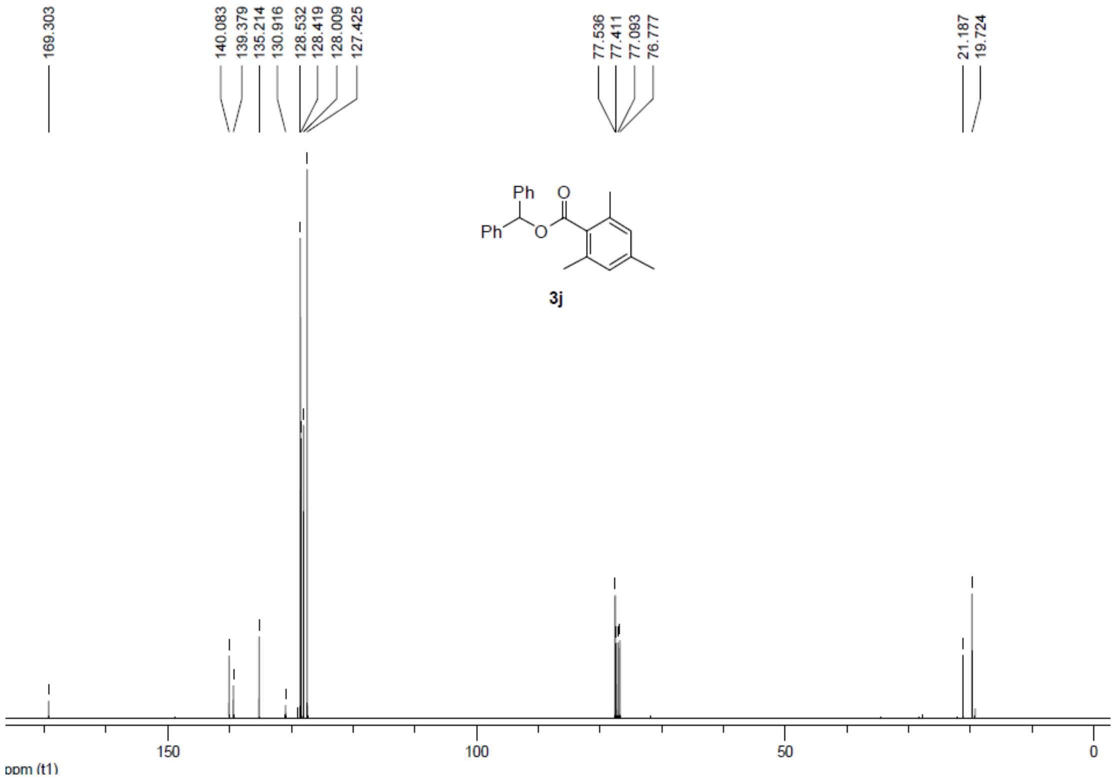
169.303
140.083
139.379
135.214
130.916
128.532
128.419
128.009
127.425
77.536
77.411
77.093
76.777
21.187
19.724
Ph
O
Ph
O
3j
150
100
50
0
ppm (t1)

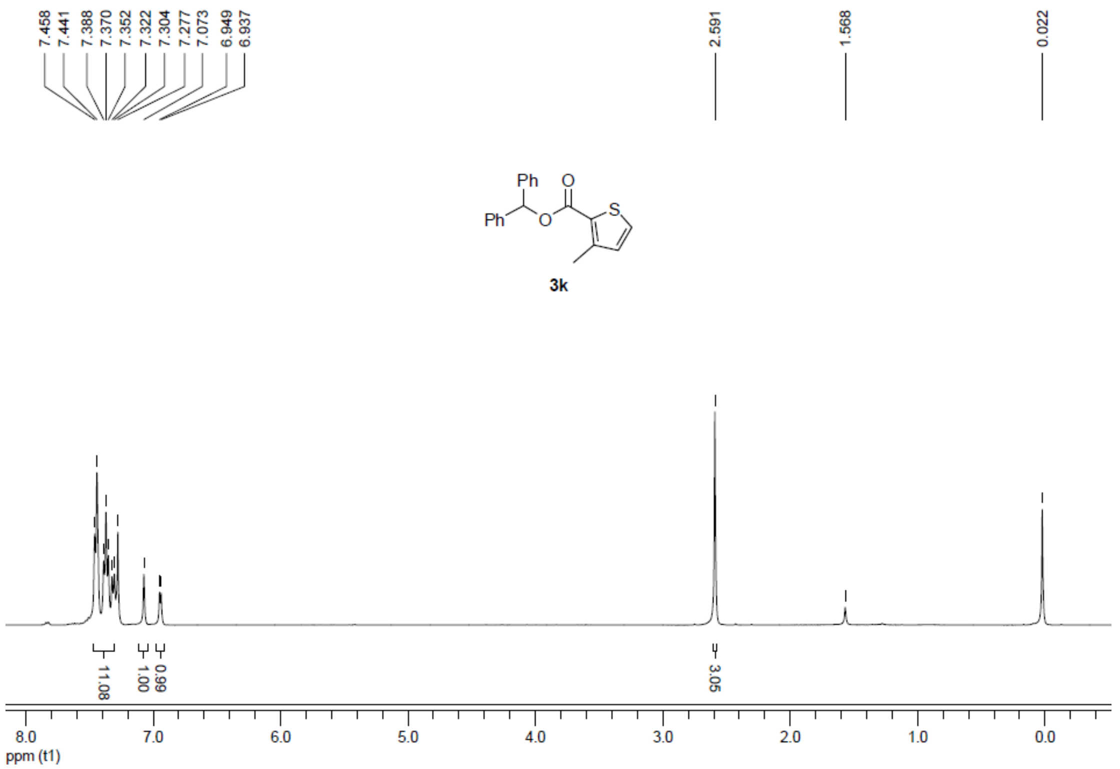
7.458
7.441
7.388
7.370
7.352
7.322
7.304
7.277
7.073
6.949
6.937
2.591
1.568
0.022
Ph
O
S
Ph
O
3k
11.08
1.00
0.99
3.05
8.0
7.0
6.0
5.0
4.0
3.0
2.0
1.0
0.0
ppm (t1)

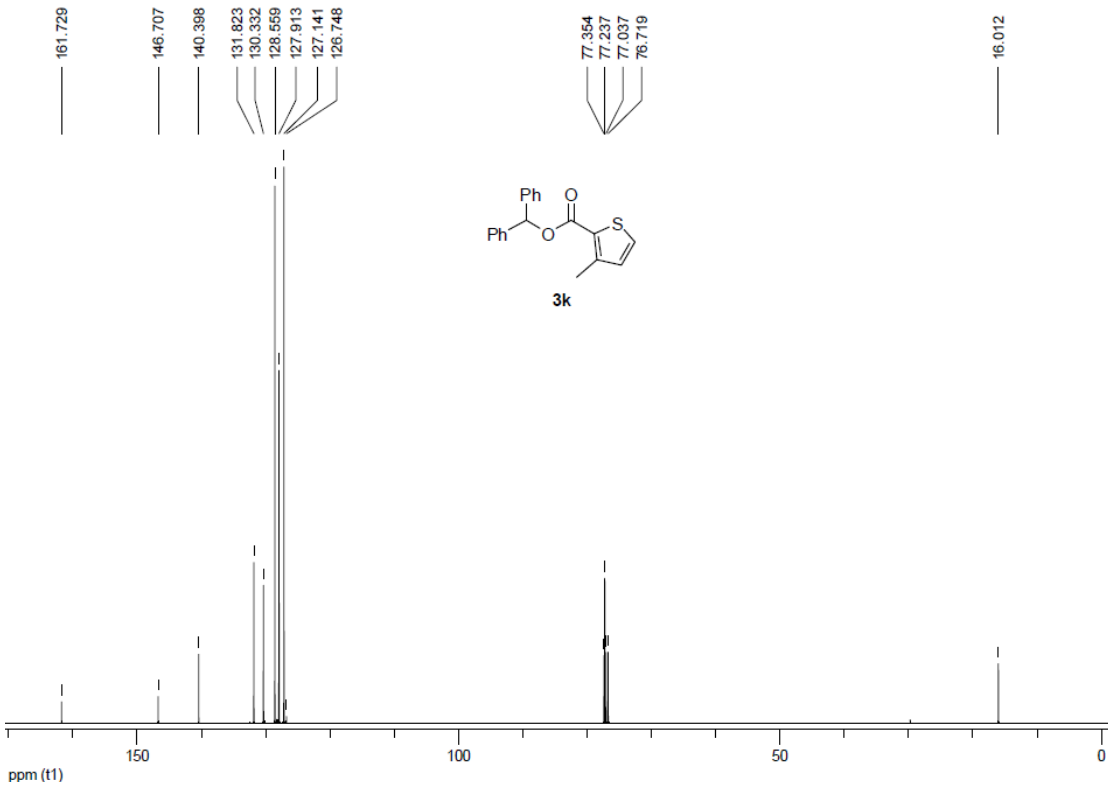
161.729
146.707
140.398
131.823
130.332
128.559
127.913
127.141
126.748
77.354
77.237
77.037
76.719
16.012
Ph
O
S
Ph
O
3k
150
100
50
0
ppm (t1)

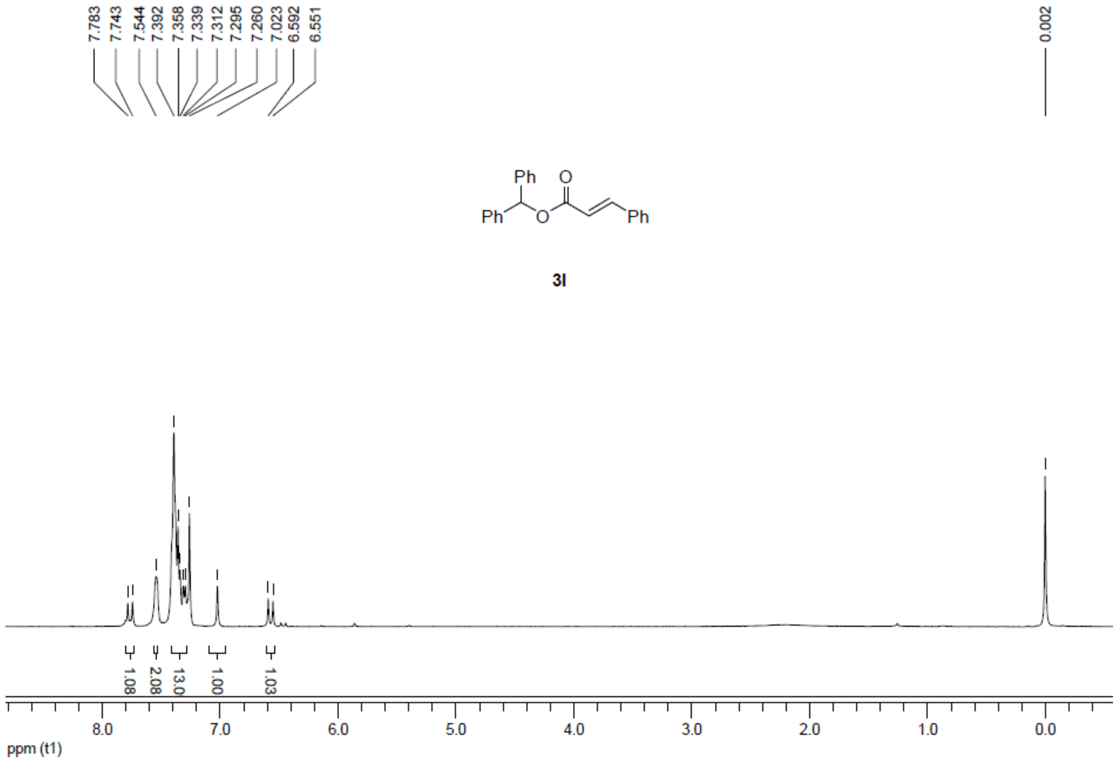
7.783
7.743
7.544
7.392
7.358
7.339
7.312
7.295
7.260
7.023
6.592
6.551
0.002
Ph
O
Ph
O
Ph
3l
1.08
2.08
13.0
1.00
1.03
8.0
7.0
6.0
5.0
4.0
3.0
2.0
1.0
0.0
ppm (t1)

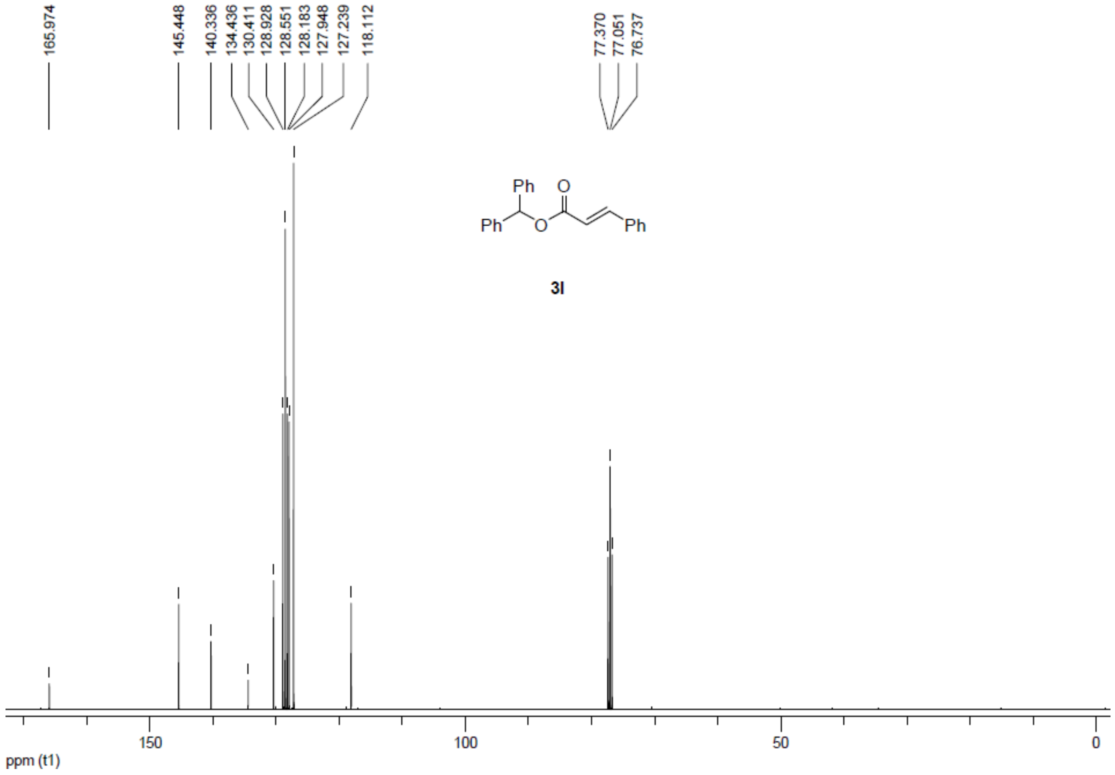
165.974
145.448
140.336
134.436
130.411
128.928
128.551
128.183
127.948
127.239
118.112
77.370
77.051
76.737
Ph
O
Ph
O
Ph
3l
150
100
50
0
ppm (t1)

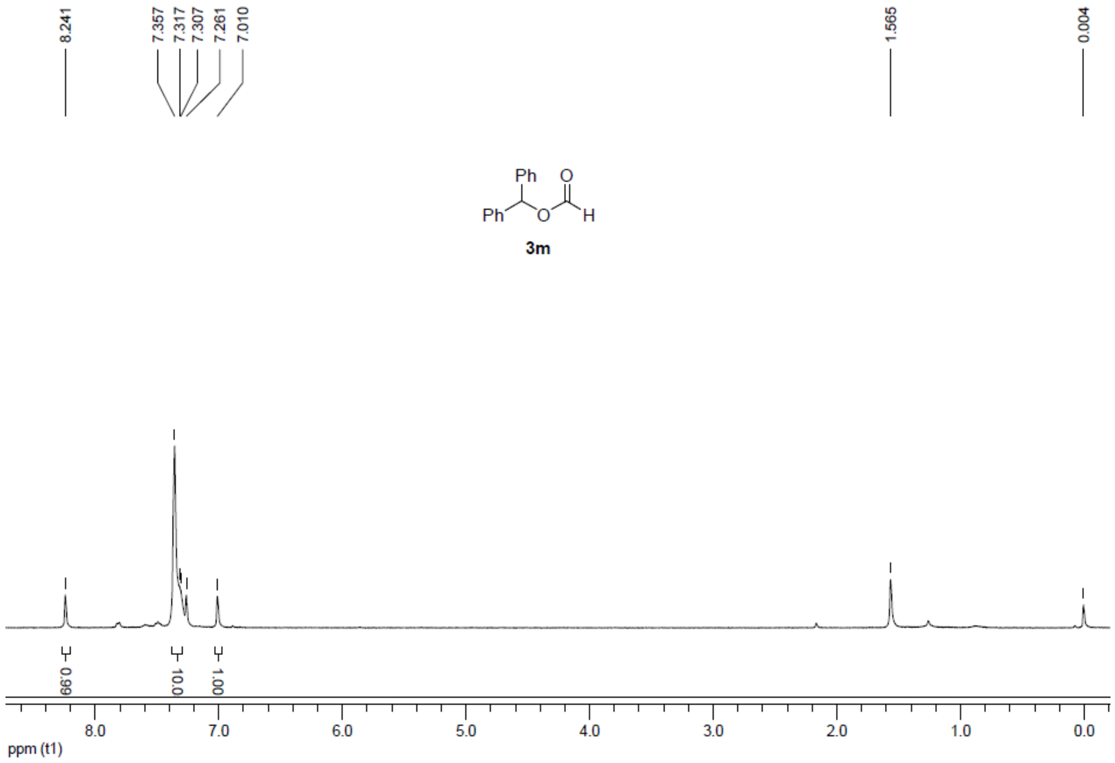

8.241
7.357
7.317
7.307
7.261
7.010
1.565
0.004
Ph
O
Ph
O
H
3m
0.99
10.0
1.00
8.0
7.0
6.0
5.0
4.0
3.0
2.0
1.0
0.0
ppm (t1)

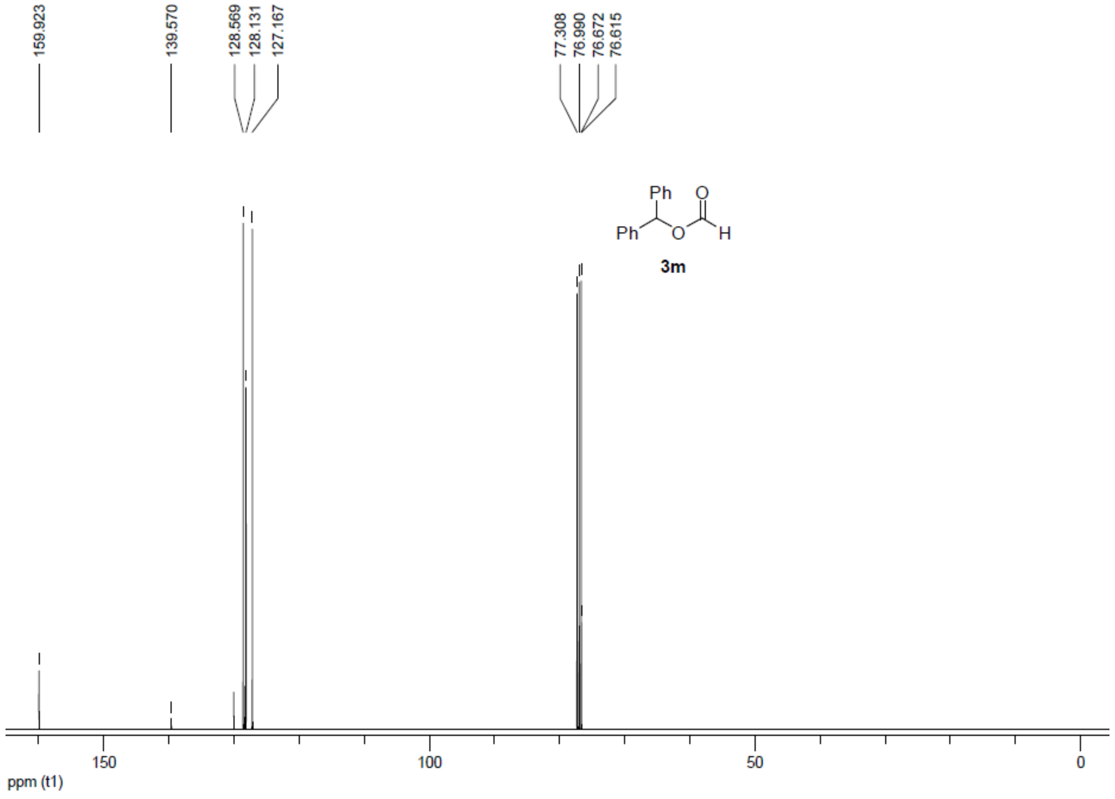

159.923
139.570
128.569
128.131
127.167
77.308
76.990
76.672
76.615
Ph
O
Ph
O
H
3m
150
100
50
0
ppm (t1)

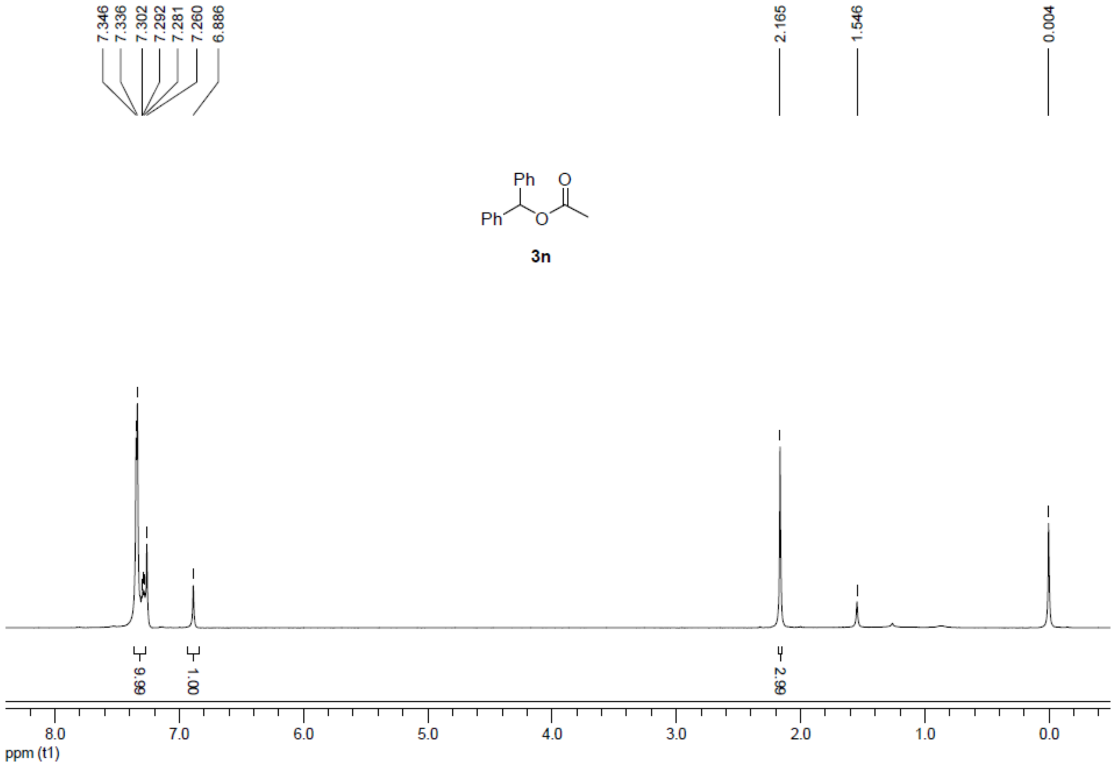
7.346
7.336
7.302
7.292
7.281
7.260
6.886
2.165
1.546
0.004
Ph
O
Ph
O
3n
9.99
1.00
2.99
8.0
7.0
6.0
5.0
4.0
3.0
2.0
1.0
0.0
ppm (t1)

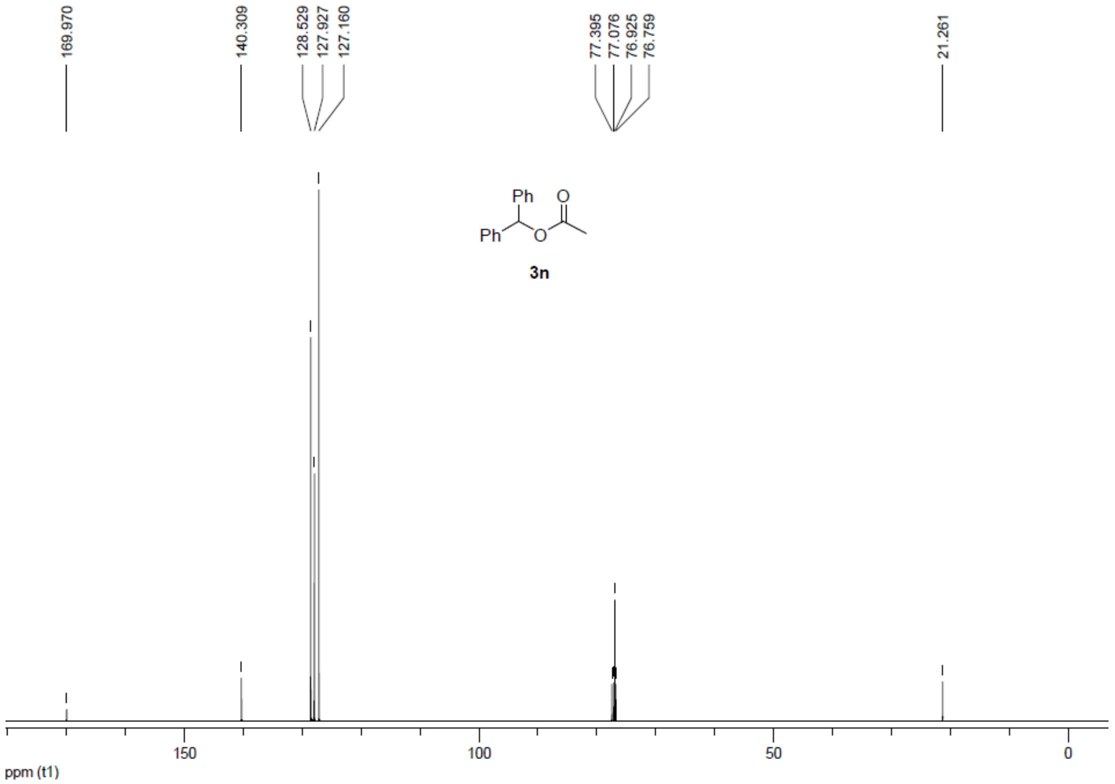
169.970
140.309
128.529
127.927
127.160
77.395
77.076
76.925
76.759
21.261
Ph
O
Ph
O
3n
150
100
50
0
ppm (t1)

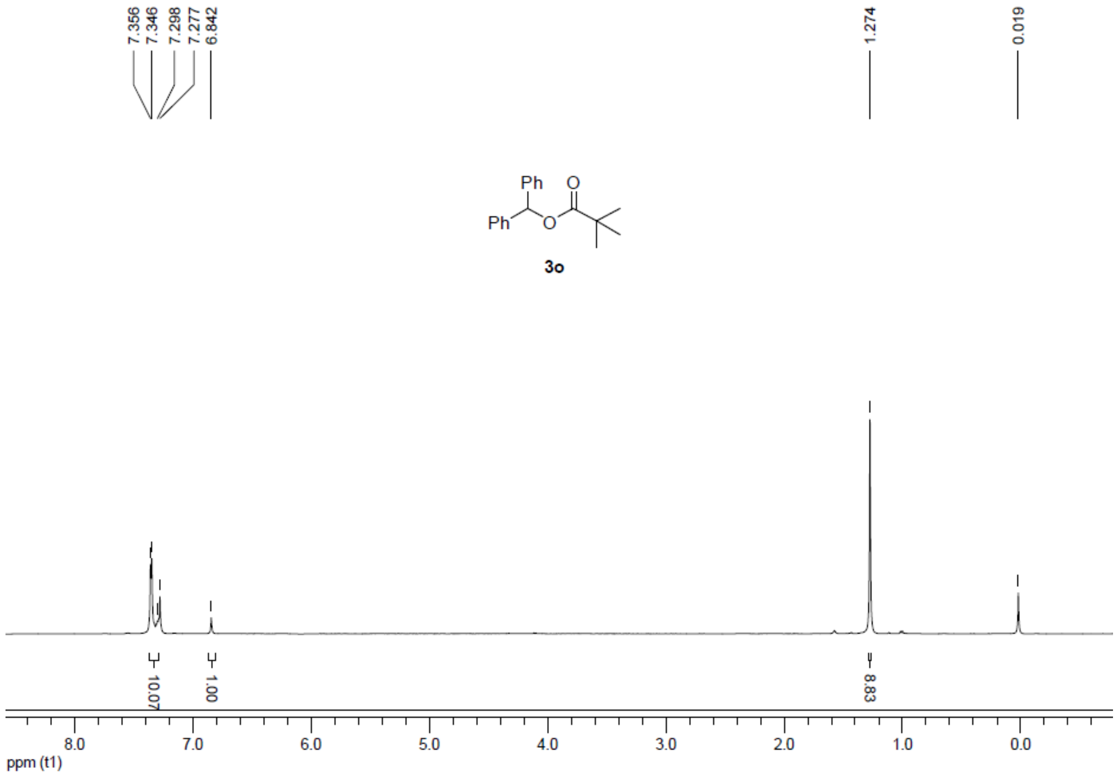
7.356
7.346
7.298
7.277
6.842
1.274
0.019
Ph
O
Ph
O
3o
10.07
1.00
8.83
8.0
7.0
6.0
5.0
4.0
3.0
2.0
1.0
0.0
ppm (t1)

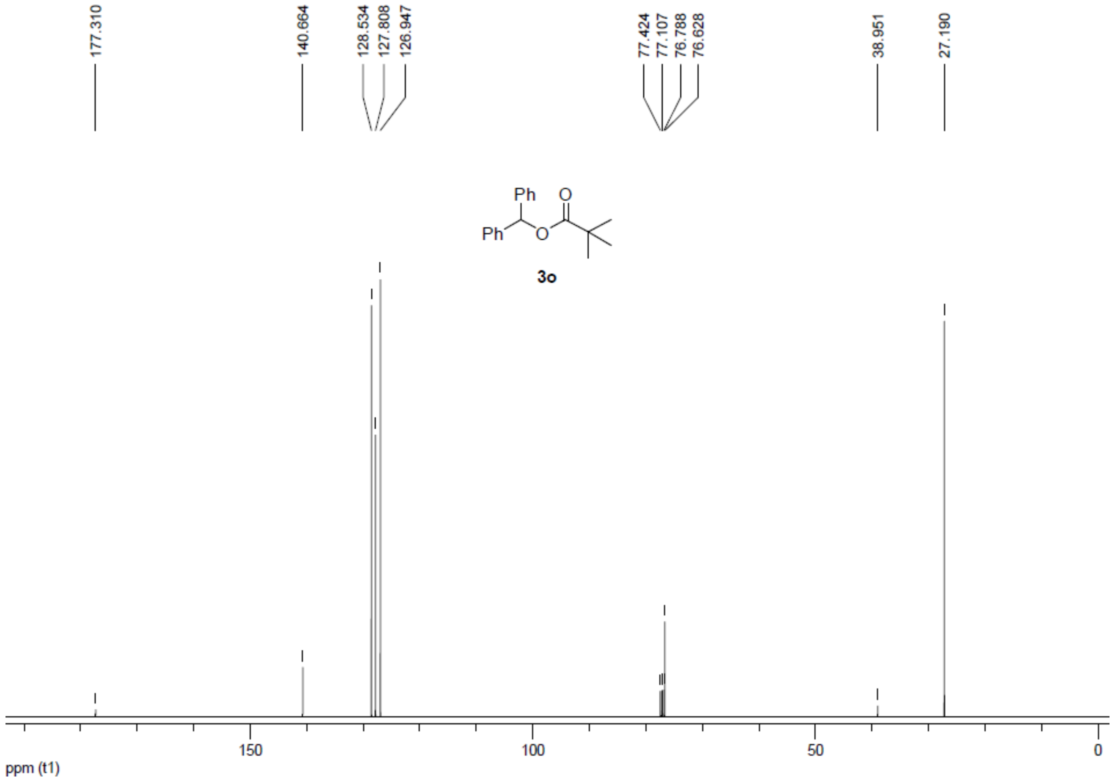
177.310
140.664
128.534
127.808
126.947
77.424
77.107
76.788
76.628
38.951
27.190
Ph
O
Ph
O
3o
150
100
50
0
ppm (t1)

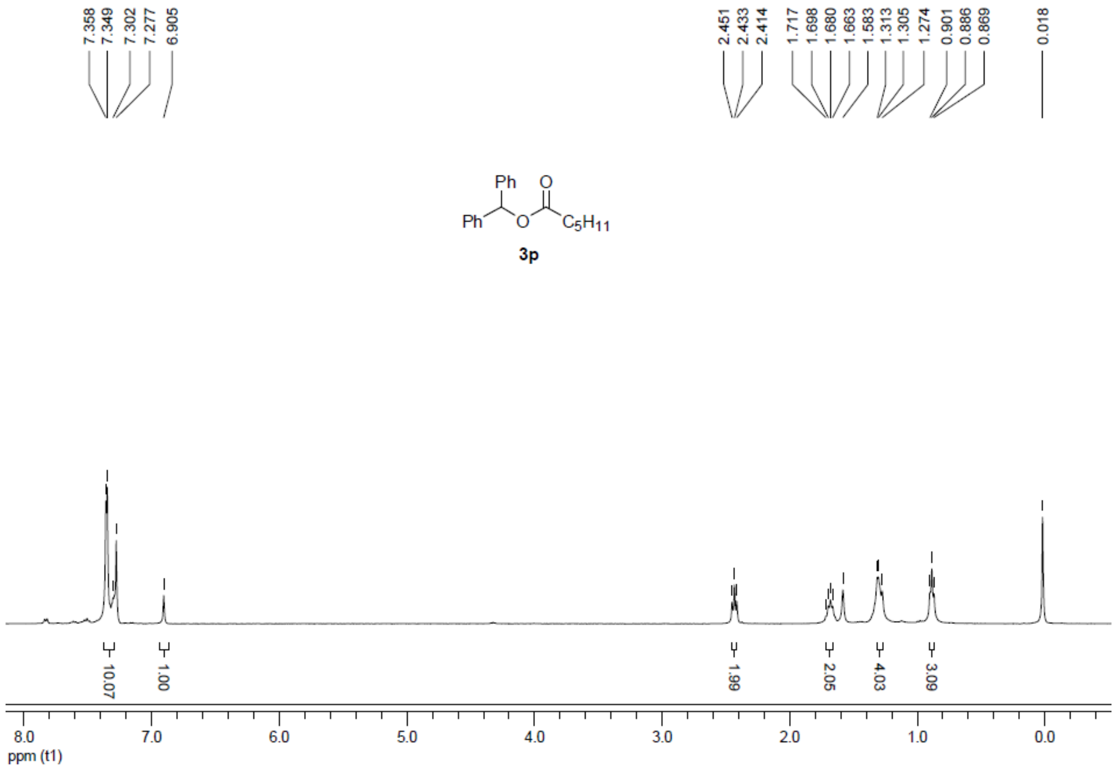
7.358
7.349
7.302
7.277
6.905
2.451
2.433
2.414
1.717
1.698
1.680
1.663
1.583
1.313
1.305
1.274
0.901
0.886
0.869
0.018
Ph
O
Ph
O
C5H11
3p
10.07
1.00
1.99
2.05
4.03
3.09
8.0
7.0
6.0
5.0
4.0
3.0
2.0
1.0
0.0
ppm (t1)

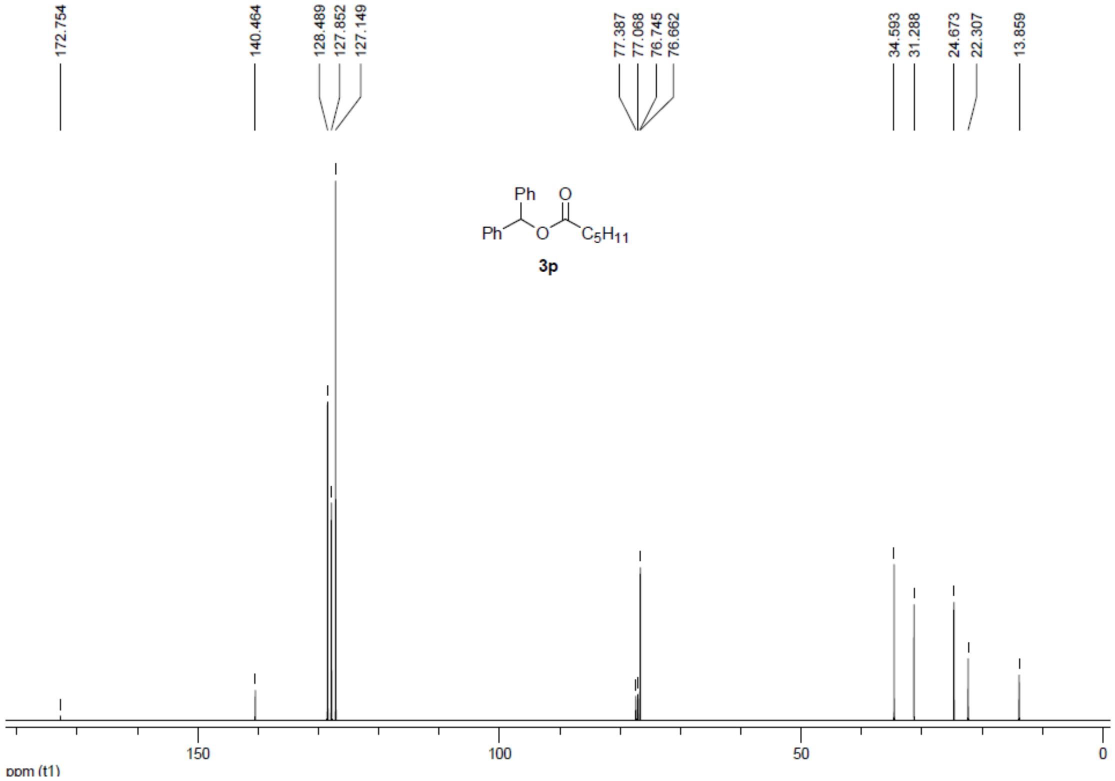
172.754
140.464
128.489
127.852
127.149
77.387
77.068
76.745
76.662
34.593
31.288
24.673
22.307
13.859
Ph
O
Ph
O
C5H11
3p
150
100
50
0
ppm (t1)

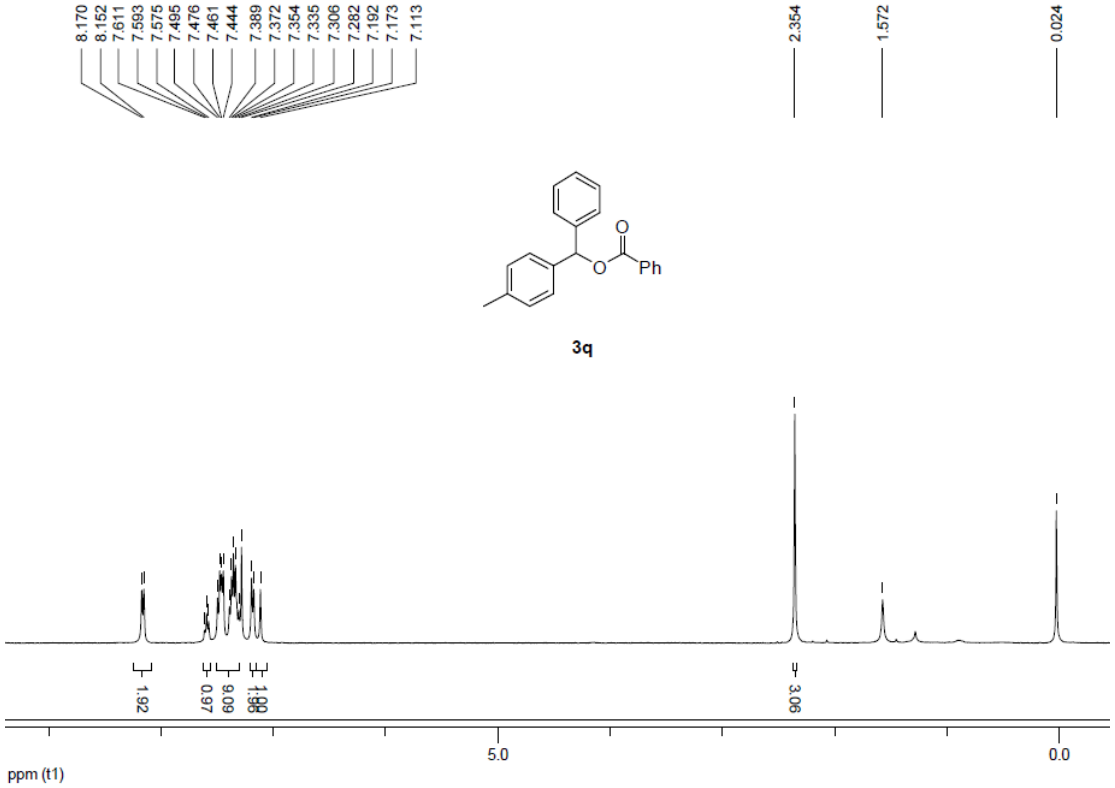
8.170
8.152
7.611
7.593
7.575
7.495
7.476
7.461
7.444
7.389
7.372
7.354
7.335
7.306
7.282
7.192
7.173
7.113
2.354
1.572
0.024
O
O
Ph
3q
1.92
0.97
9.09
3.06
5.0
0.0
ppm (t1)

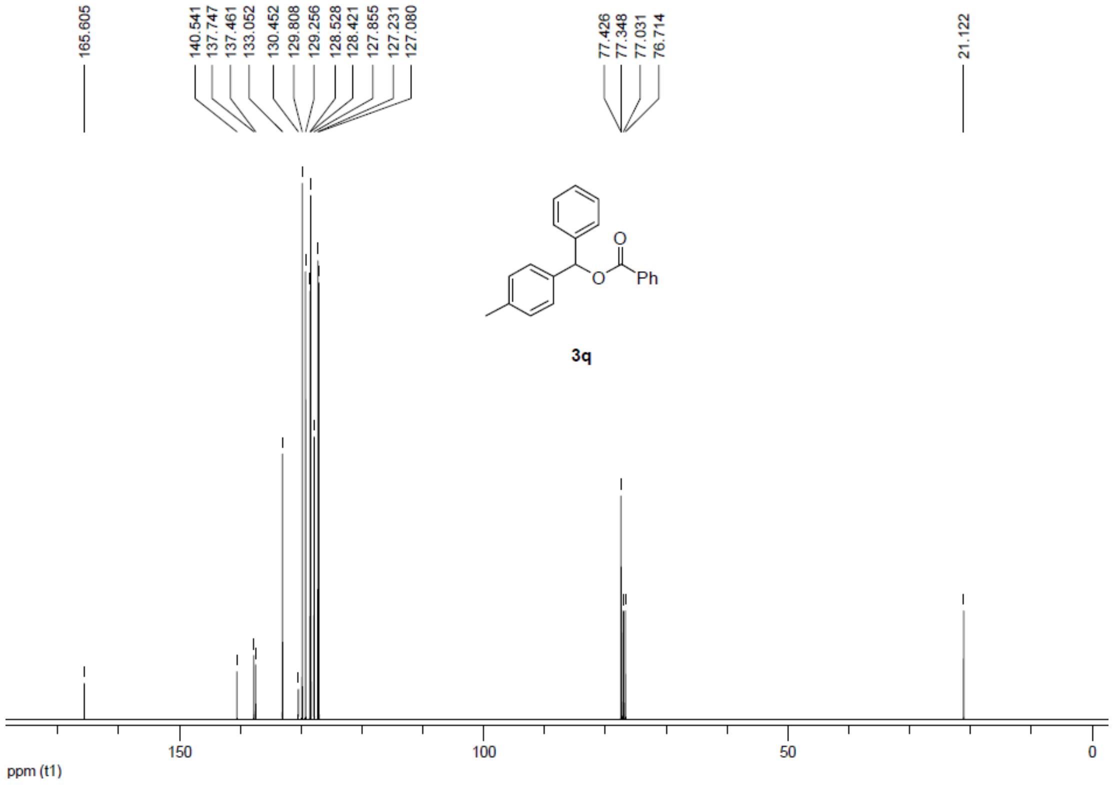
165.605
140.541
137.747
137.461
133.052
130.452
129.808
129.256
128.528
128.421
127.855
127.231
127.080
77.426
77.348
77.031
76.714
21.122
O
O
Ph
3q
150
100
50
0
ppm (t1)

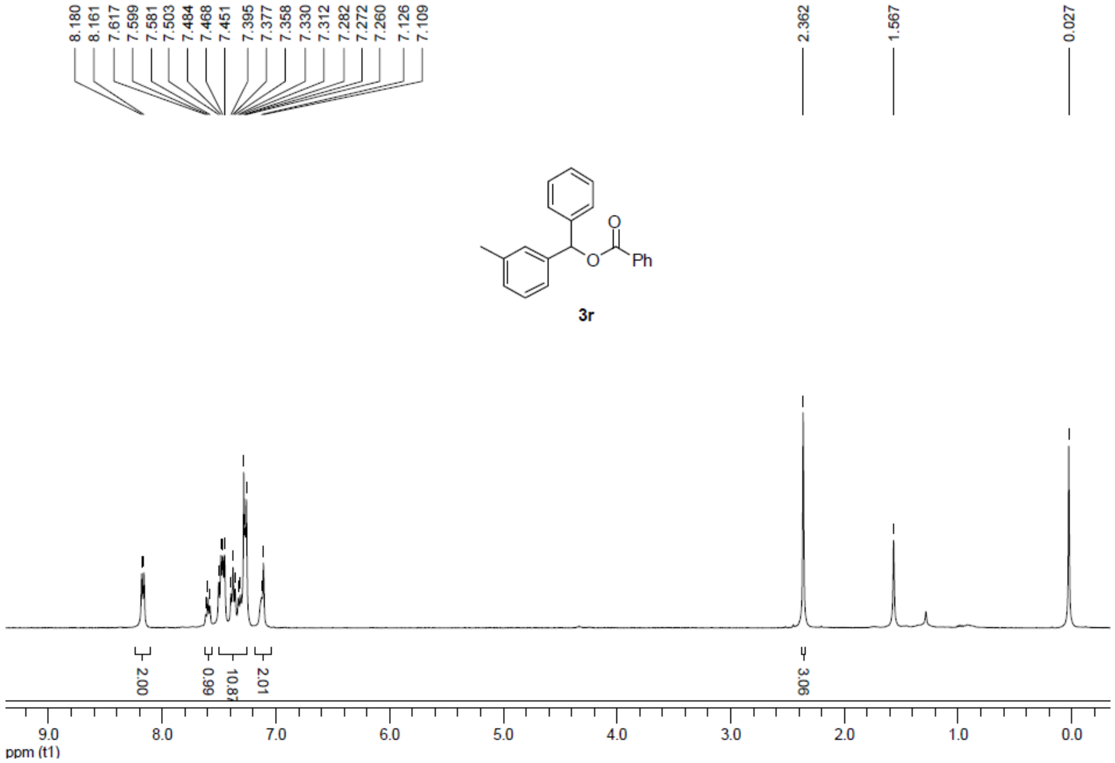
8.180
8.161
7.617
7.599
7.581
7.503
7.484
7.468
7.451
7.395
7.377
7.358
7.330
7.312
7.282
7.272
7.260
7.126
7.109
2.362
1.567
0.027
O
Ph
3r
2.00
0.99
10.87
2.01
3.06
9.0
8.0
7.0
6.0
5.0
4.0
3.0
2.0
1.0
0.0
ppm (t1)

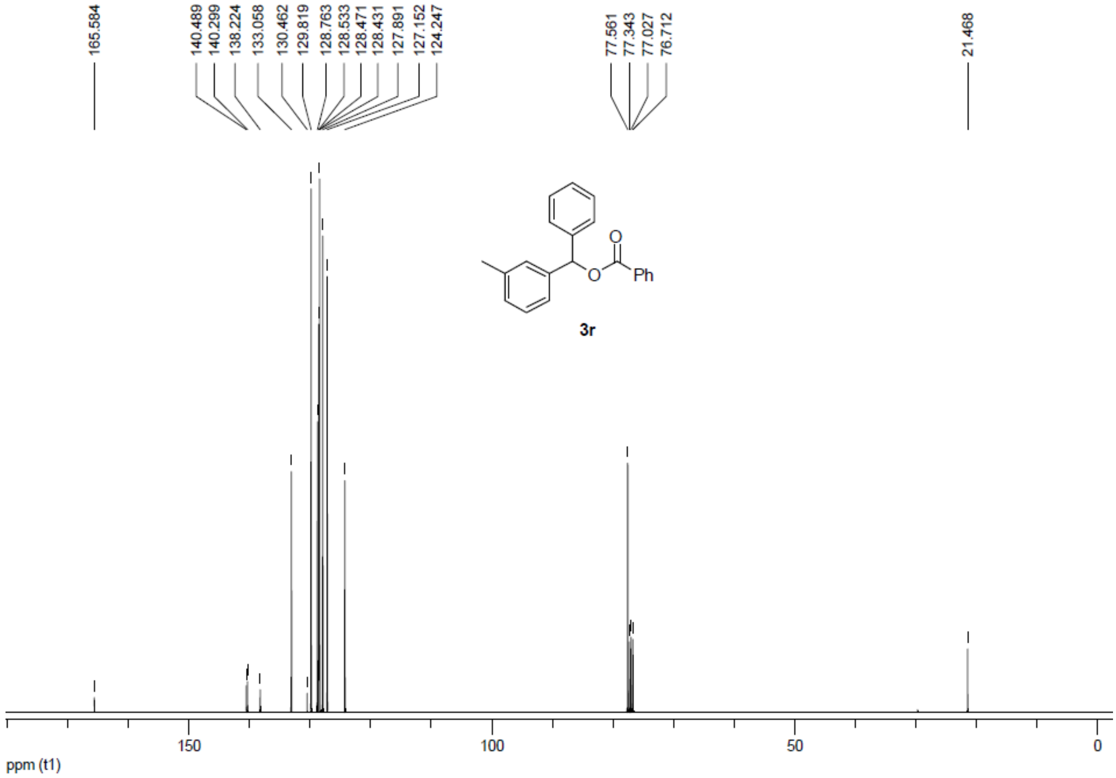
165.584
140.489
140.299
138.224
133.058
130.462
129.819
128.763
128.533
128.471
128.431
127.891
127.152
124.247
77.561
77.343
77.027
76.712
21.468
O
Ph
3r
150
100
50
0
ppm (t1)

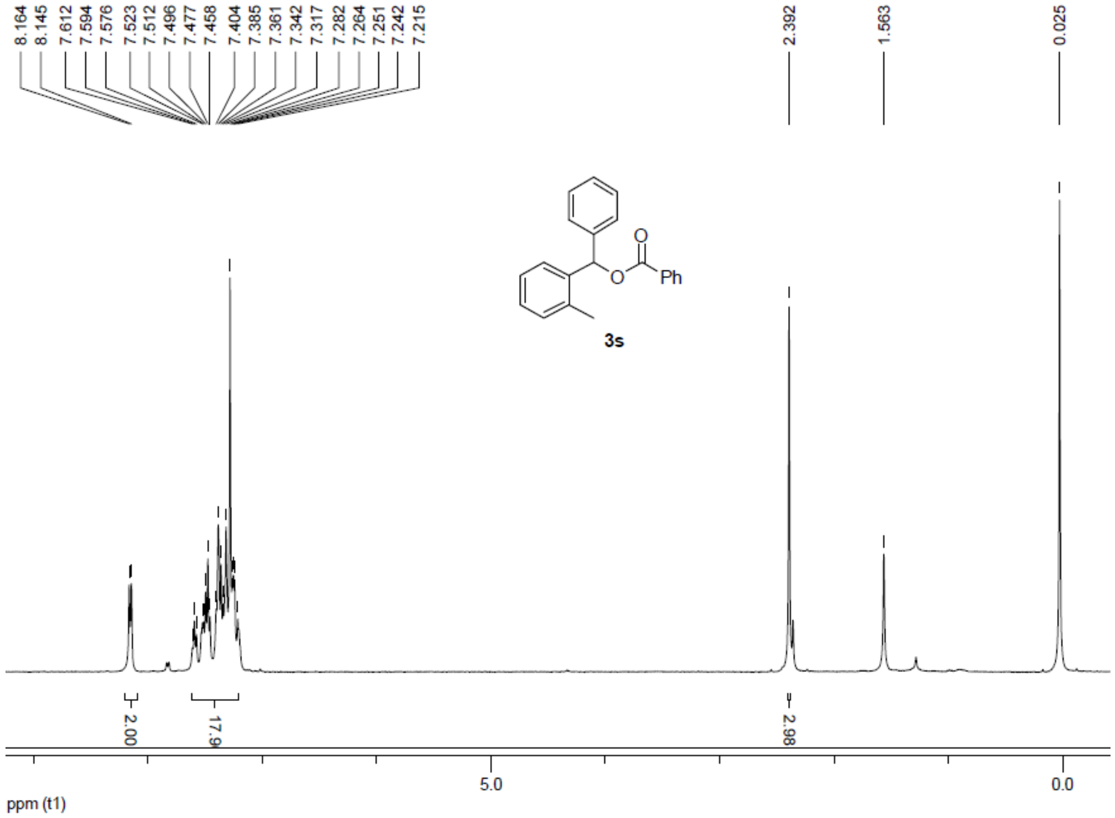
8.164
8.145
7.612
7.594
7.576
7.523
7.512
7.496
7.477
7.458
7.404
7.385
7.361
7.342
7.317
7.282
7.264
7.251
7.242
7.215
2.392
1.563
0.025
O
O
Ph
3s
2.00
17.9
2.98
5.0
0.0
ppm (t1)

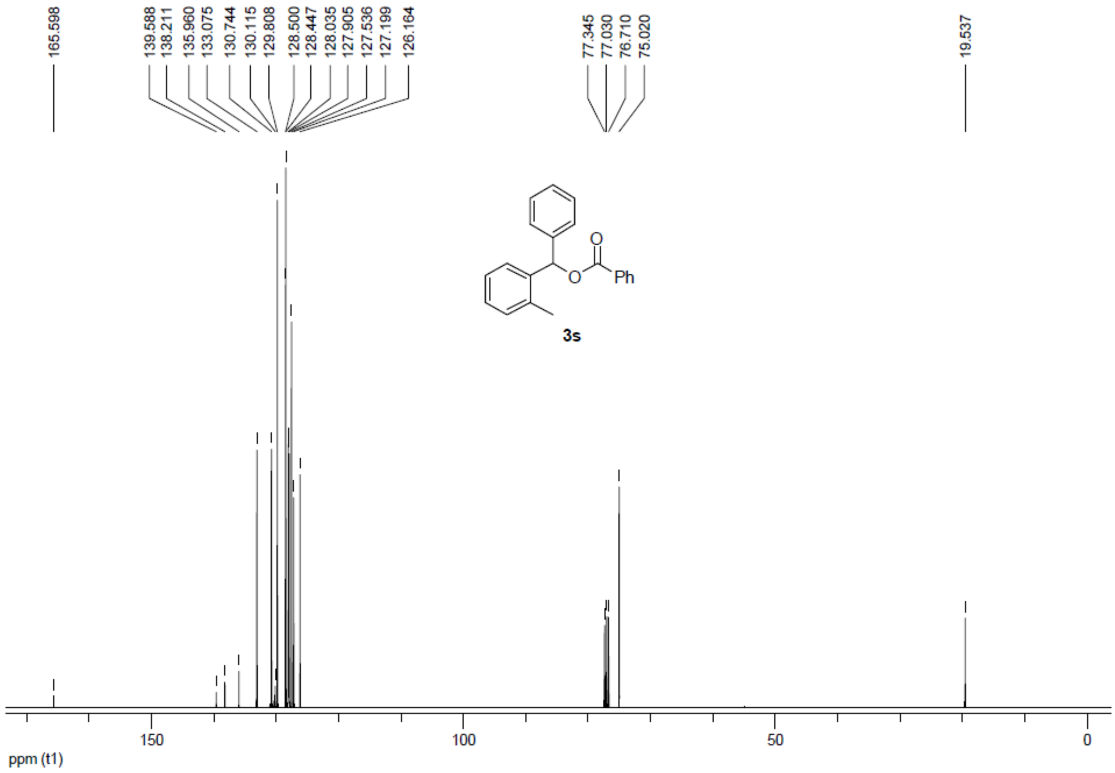
165.598
139.588
138.211
135.960
133.075
130.744
130.115
129.808
128.500
128.447
128.035
127.905
127.536
127.199
126.164
77.345
77.030
76.710
75.020
19.537
O
O
Ph
3s
150
100
50
0
ppm (t1)

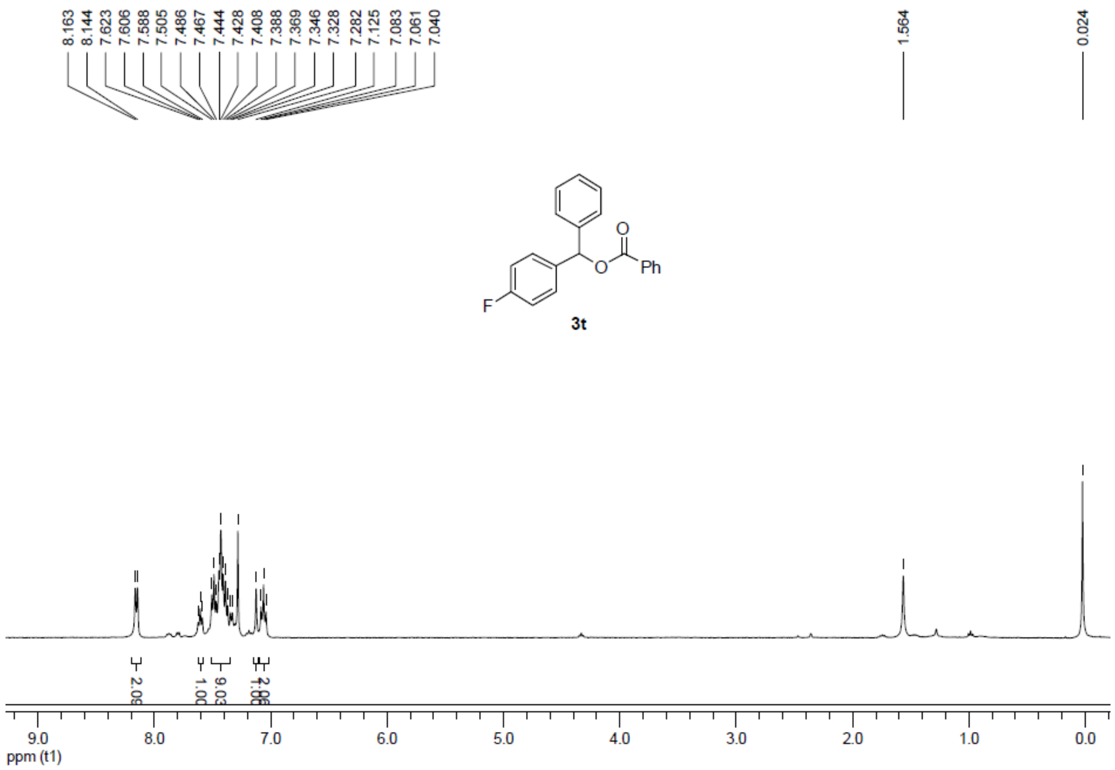
8.163
8.144
7.623
7.606
7.588
7.505
7.486
7.467
7.444
7.428
7.408
7.388
7.369
7.346
7.328
7.282
7.125
7.083
7.061
7.040
1.564
0.024
O
O
Ph
F
3t
1.00
9.03
9.0
8.0
7.0
6.0
5.0
4.0
3.0
2.0
1.0
0.0
ppm (t1)

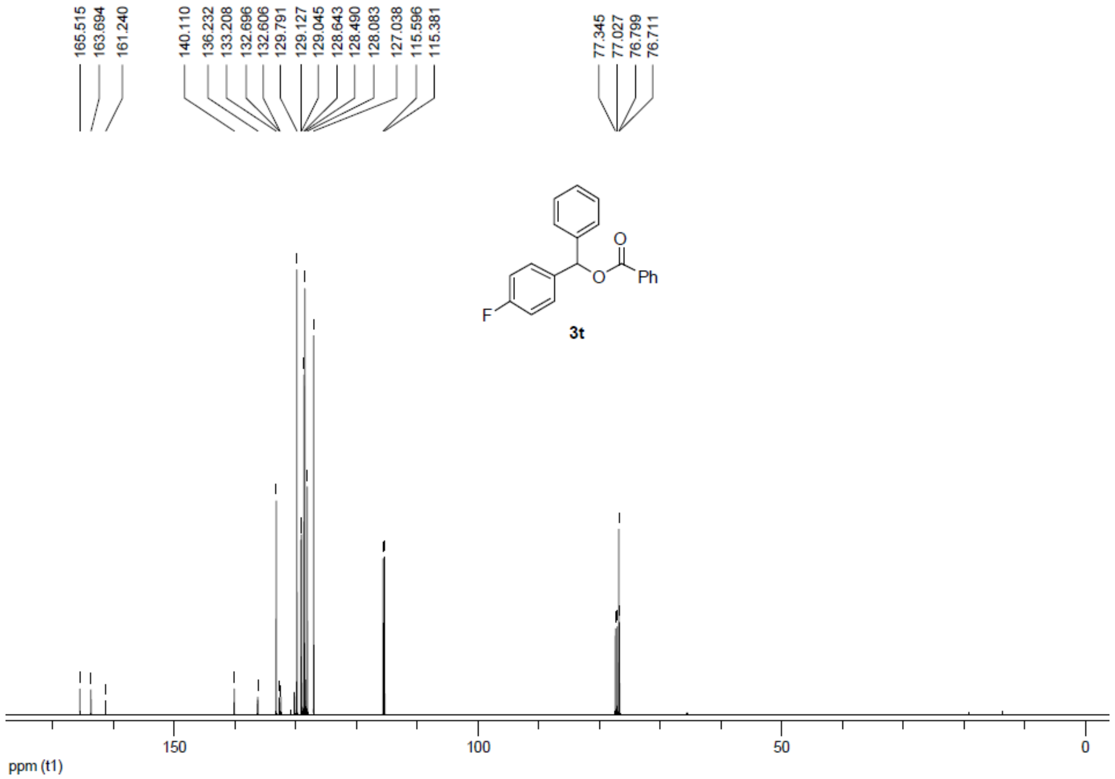
165.515
163.694
161.240
140.110
136.232
133.208
132.696
132.606
129.791
129.127
129.045
128.643
128.490
128.083
127.038
115.596
115.381
77.345
77.027
76.799
76.711
O
O
Ph
F
3t
150
100
50
0
ppm (t1)

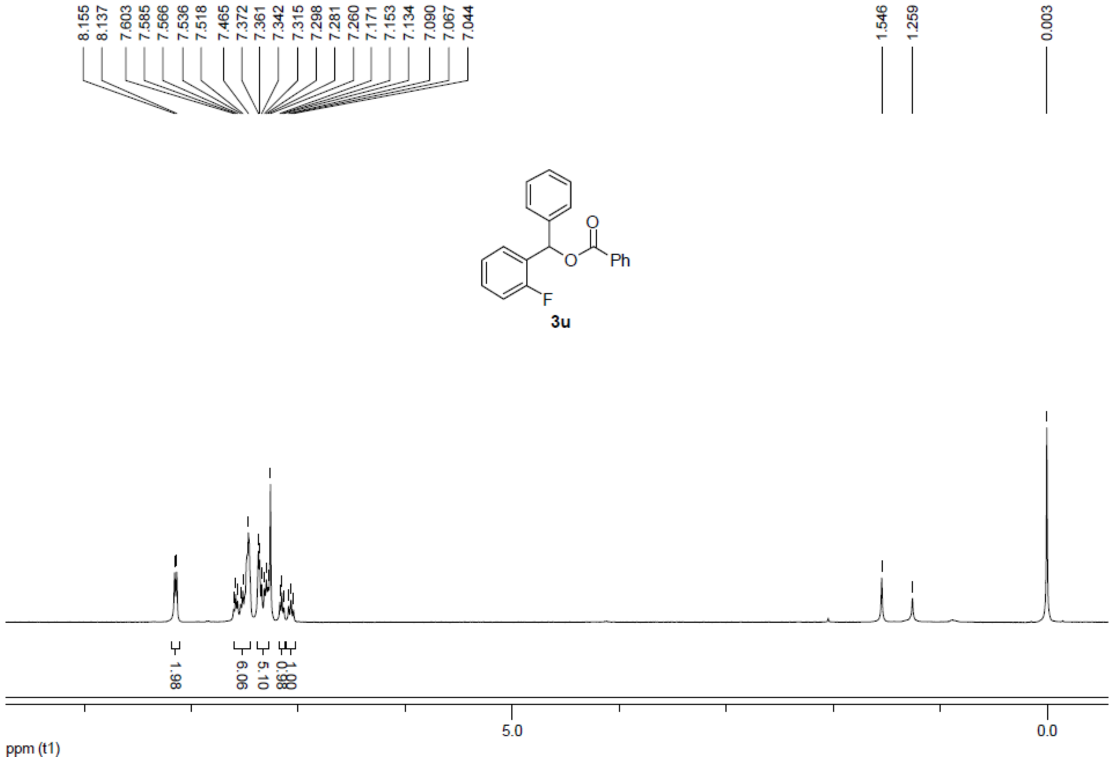
8.155
8.137
7.603
7.585
7.566
7.536
7.518
7.465
7.372
7.361
7.342
7.315
7.298
7.281
7.260
7.171
7.153
7.134
7.090
7.067
7.044
1.546
1.259
0.003
O
O
Ph
F
3u
1.98
6.06
5.10
5.0
0.0
ppm (t1)

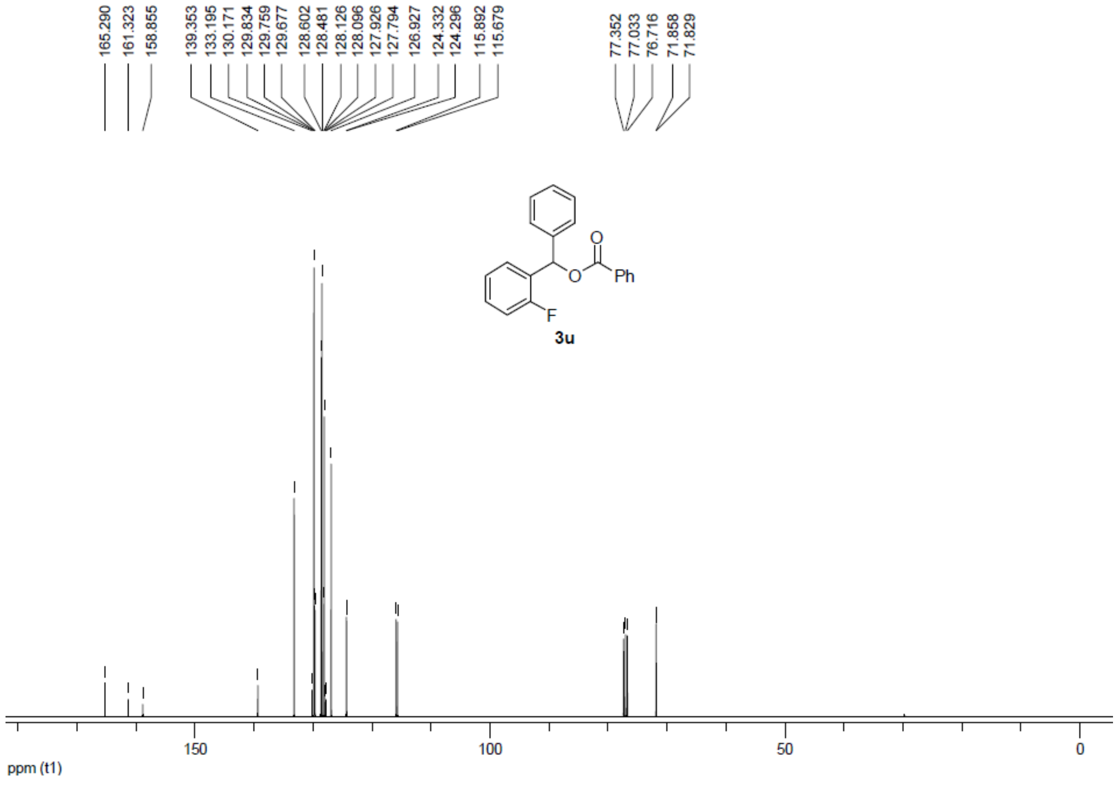
165.290
161.323
158.855
139.353
133.195
130.171
129.834
129.759
129.677
128.602
128.481
128.126
128.096
127.926
127.794
126.927
124.332
124.296
115.892
115.679
77.352
77.033
76.716
71.858
71.829
O
O
Ph
F
3u
150
100
50
0
ppm (t1)

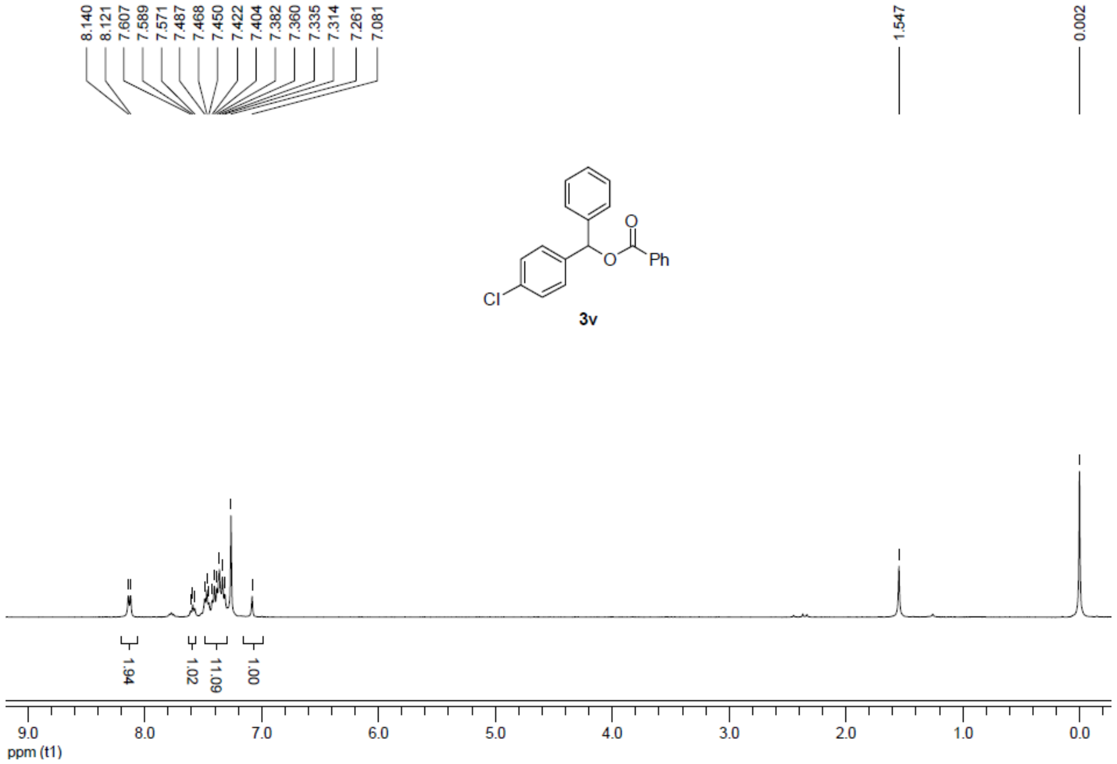
8.140 8.121 7.607 7.589 7.571 7.487 7.468 7.450 7.422 7.404 7.382 7.360 7.335 7.314 7.261 7.081
1.547
0.002
O
Ph
O
Cl
3v
1.94
1.02
11.09
1.00
9.0 8.0 7.0 6.0 5.0 4.0 3.0 2.0 1.0 0.0
ppm (t1)

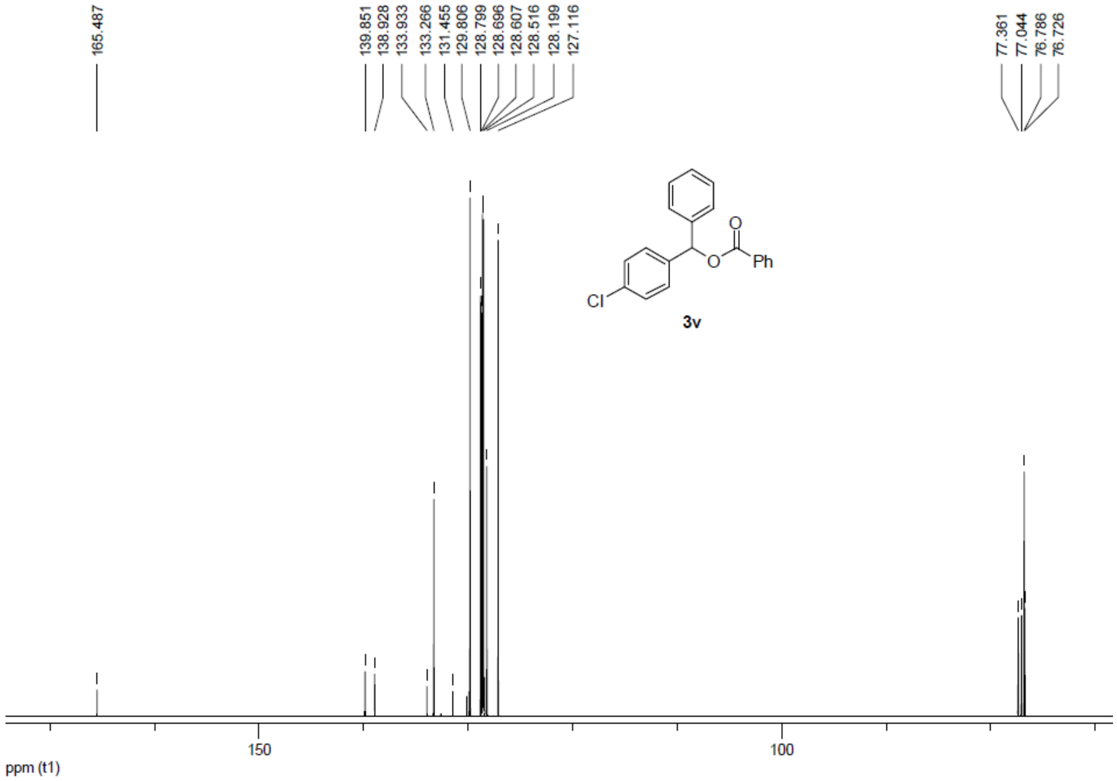
165.487
139.851 138.928 133.933 133.266 131.455 129.806 128.799 128.696 128.607 128.516 128.199 127.116
77.361 77.044 76.786 76.726
O
Ph
O
Cl
3v
150
100
ppm (t1)

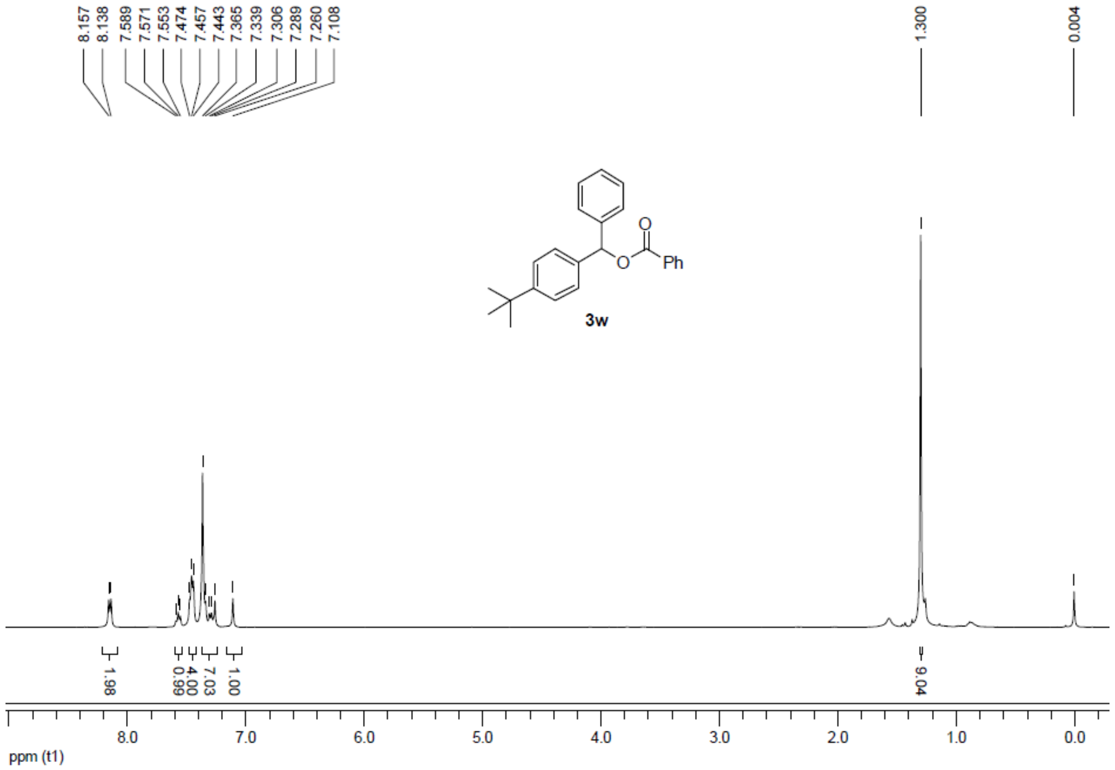
8.157
8.138
7.589
7.571
7.553
7.474
7.457
7.443
7.365
7.339
7.306
7.289
7.260
7.108
1.300
0.004
O
O
Ph
3w
1.98
0.99
4.00
7.03
1.00
9.04
8.0
7.0
6.0
5.0
4.0
3.0
2.0
1.0
0.0
ppm (t1)

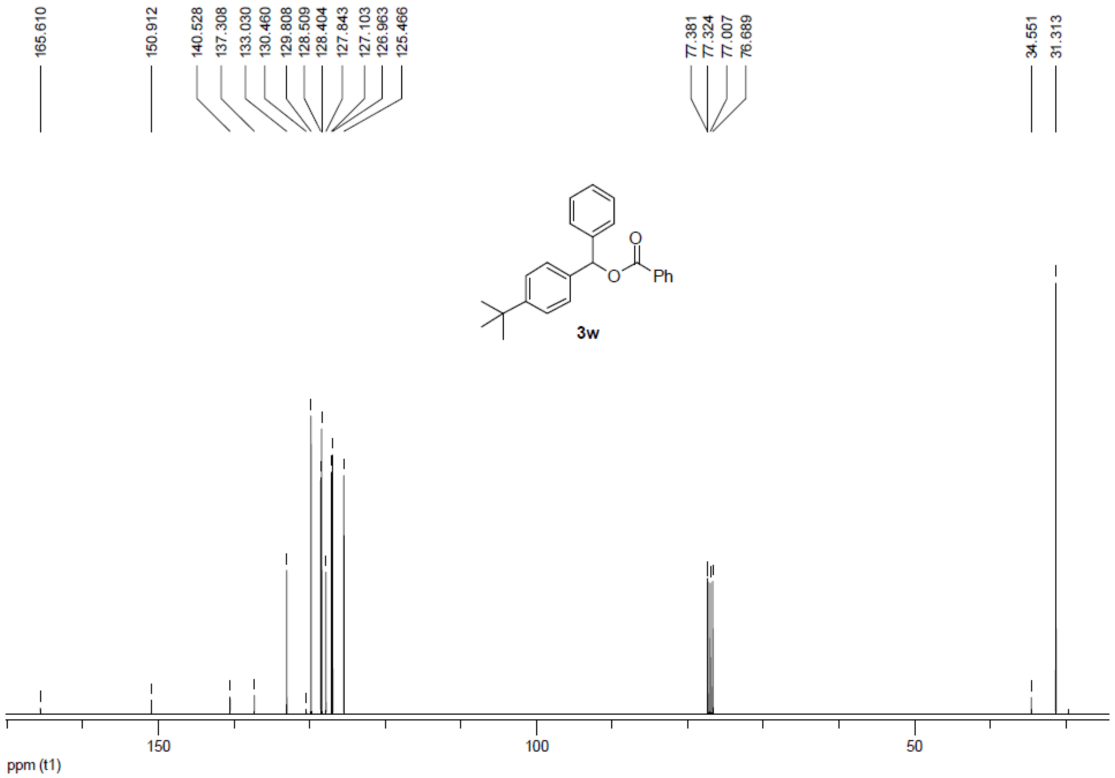
165.610
150.912
140.528
137.308
133.030
130.460
129.808
128.509
128.404
127.843
127.103
126.963
125.466
77.381
77.324
77.007
76.689
34.551
31.313
O
O
Ph
3w
150
100
50
ppm (t1)

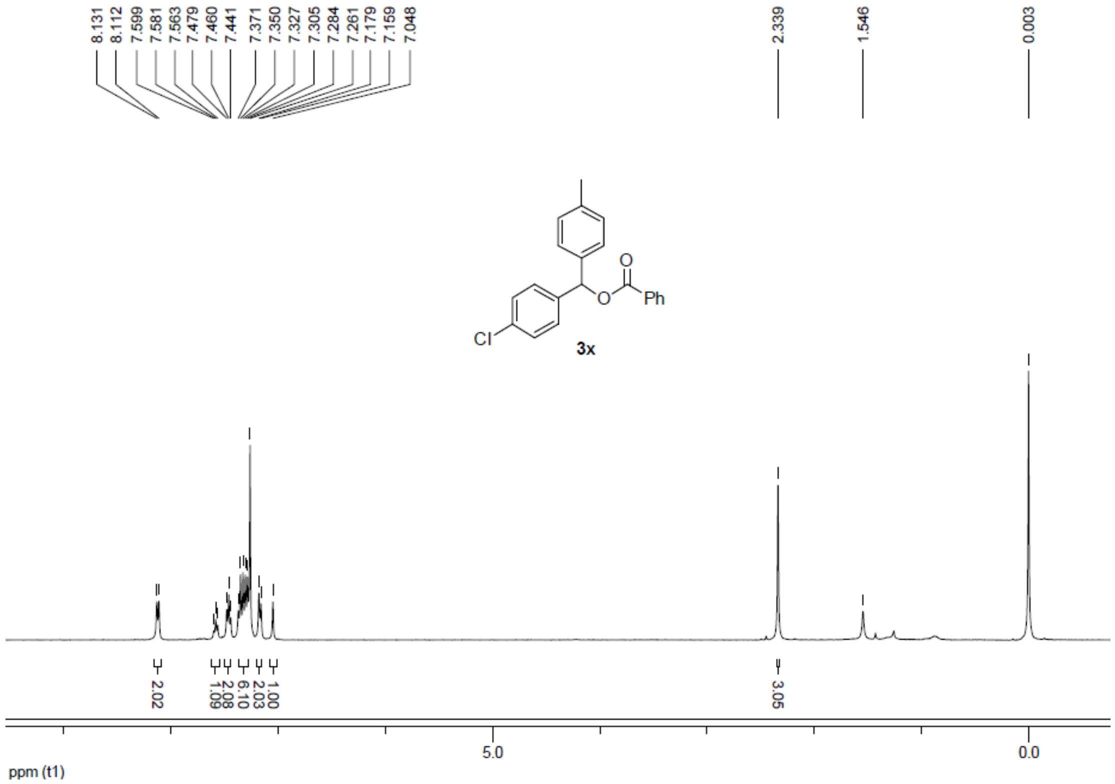

8.131
8.112
7.599
7.581
7.563
7.479
7.460
7.441
7.371
7.350
7.327
7.305
7.284
7.261
7.179
7.159
7.048
2.339
1.546
0.003
O
Ph
Cl
3x
2.02
1.09
2.08
6.10
2.03
1.00
3.05
5.0
0.0
ppm (t1)

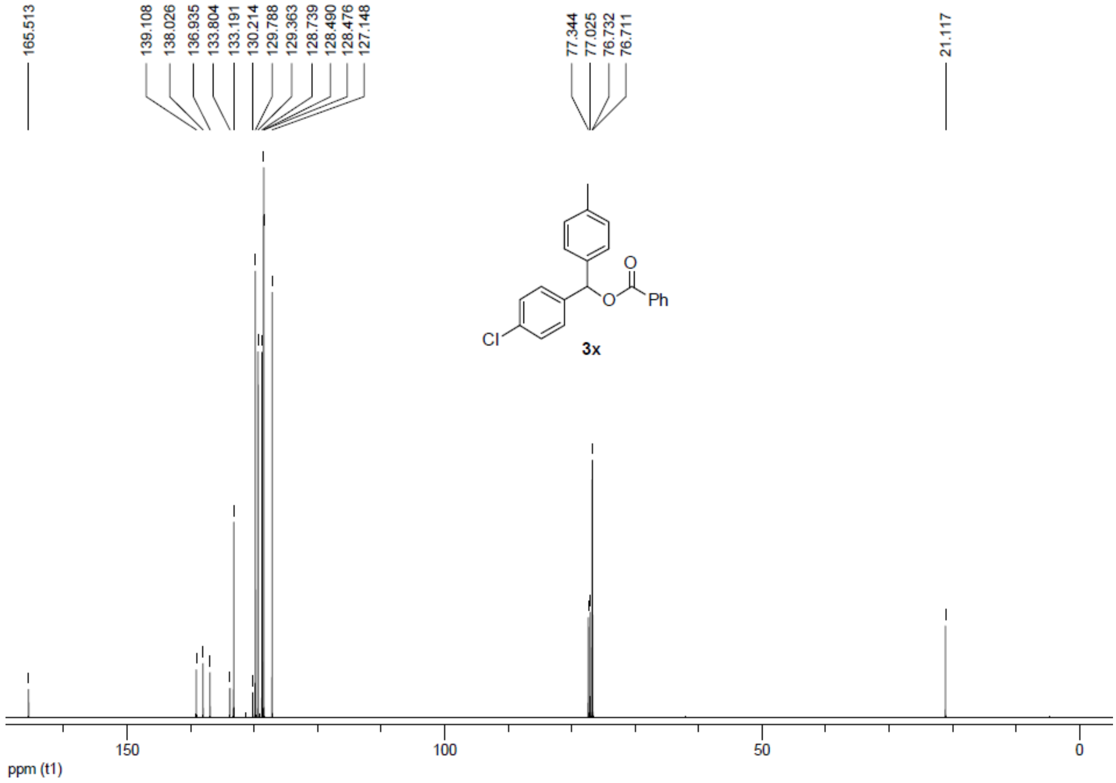

165.513
139.108
138.026
136.935
133.804
133.191
130.214
129.788
129.363
128.739
128.490
128.476
127.148
77.344
77.025
76.732
76.711
21.117
O
Ph
Cl
3x
150
100
50
0
ppm (t1)

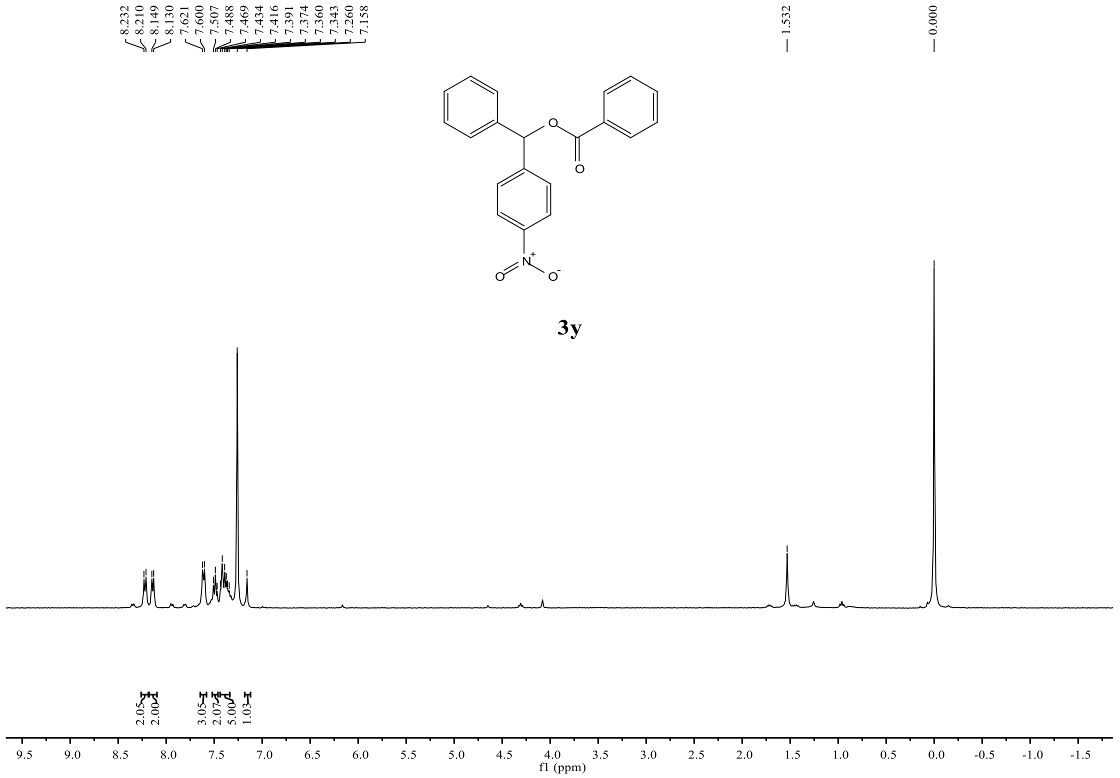
8.232
8.210
8.149
8.130
7.621
7.600
7.507
7.488
7.469
7.434
7.416
7.391
7.374
7.360
7.343
7.260
7.158
1.532
0.000
3y
2.05
2.00
3.05
2.07
5.00
1.03
9.5 9.0 8.5 8.0 7.5 7.0 6.5 6.0 5.5 5.0 4.5 4.0 3.5 3.0 2.5 2.0 1.5 1.0 0.5 0.0 -0.5 -1.0 -1.5
f1 (ppm)

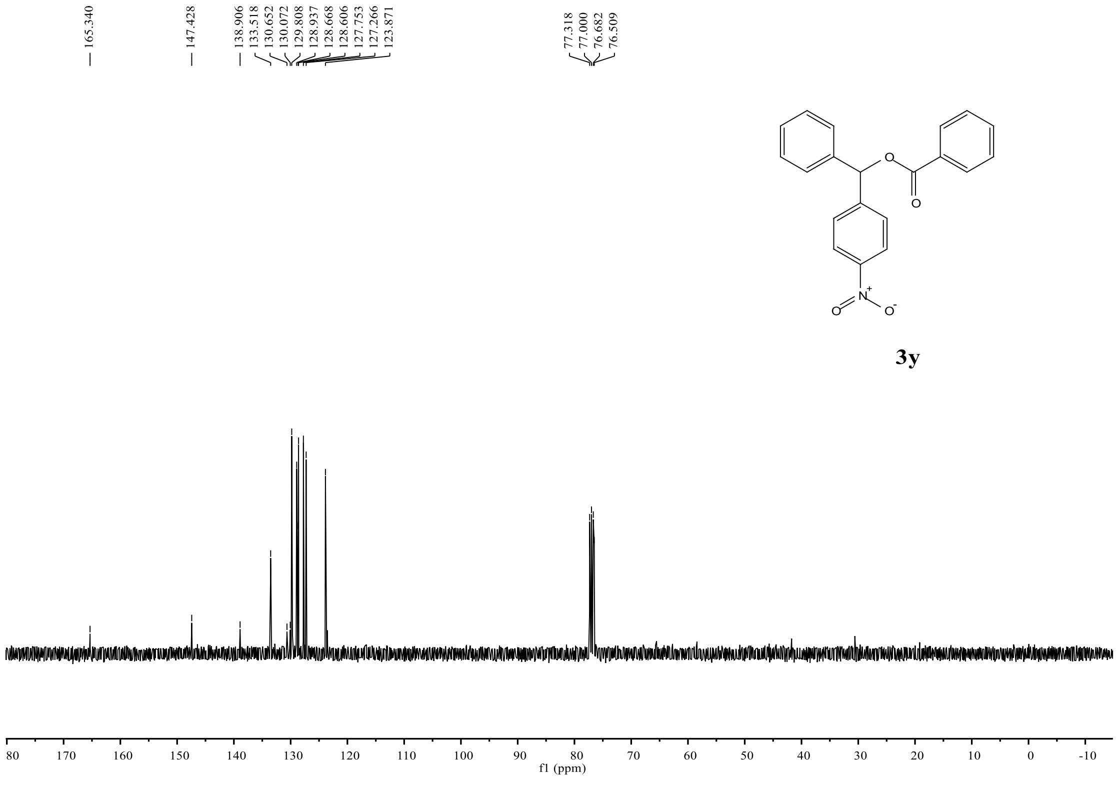
165.340
147.428
138.906
133.518
130.652
130.072
129.808
128.937
128.668
128.606
127.753
127.266
123.871
77.318
77.000
76.682
76.509
3y
80 170 160 150 140 130 120 110 100 90 80 70 60 50 40 30 20 10 0 -10
f1 (ppm)

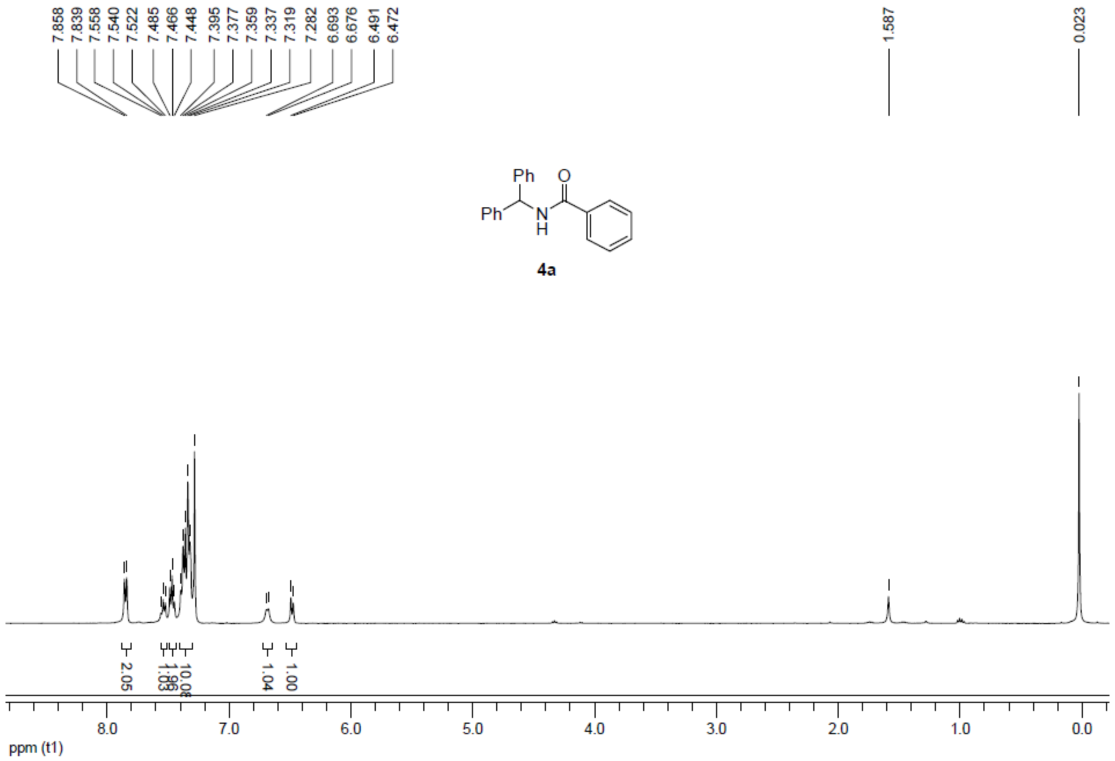
7.858
7.839
7.558
7.540
7.522
7.485
7.466
7.448
7.395
7.377
7.359
7.337
7.319
7.282
6.693
6.676
6.491
6.472
1.587
0.023
Ph
O
Ph
N
H
4a
2.05
1.03
1.05
10.08
1.04
1.00
8.0
7.0
6.0
5.0
4.0
3.0
2.0
1.0
0.0
ppm (t1)

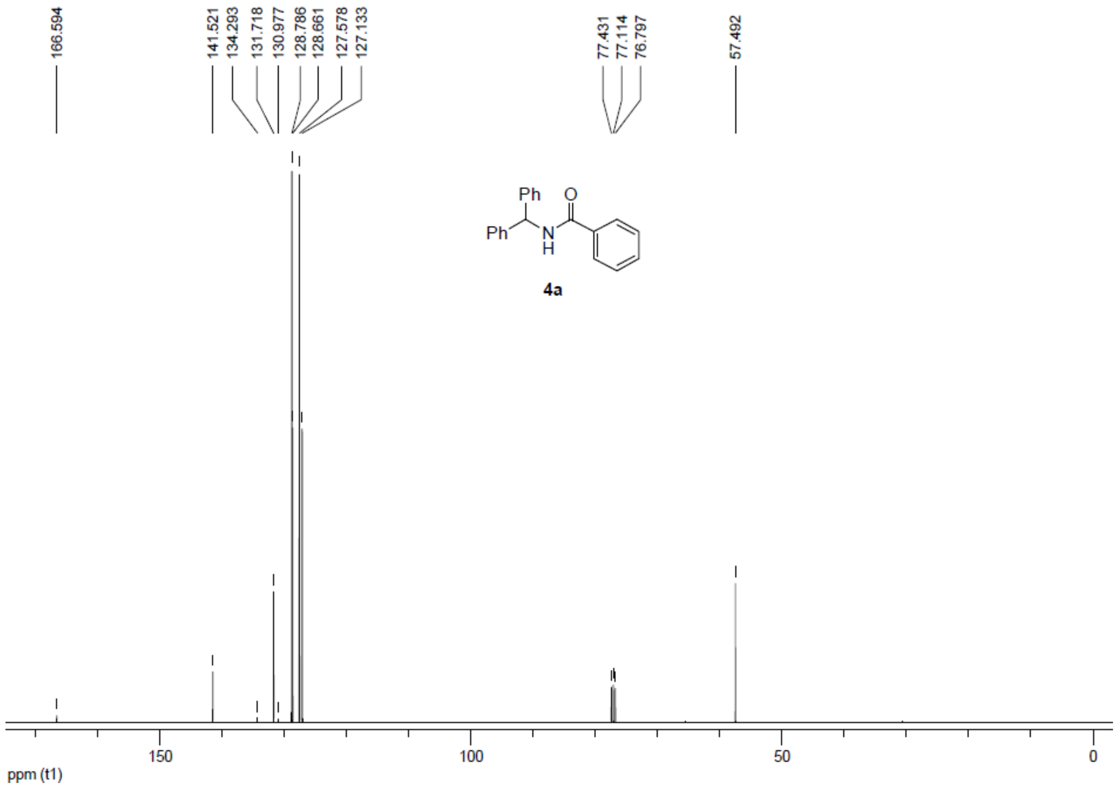
166.594
141.521
134.293
131.718
130.977
128.786
128.661
127.578
127.133
77.431
77.114
76.797
57.492
Ph
O
Ph
N
H
4a
150
100
50
0
ppm (t1)

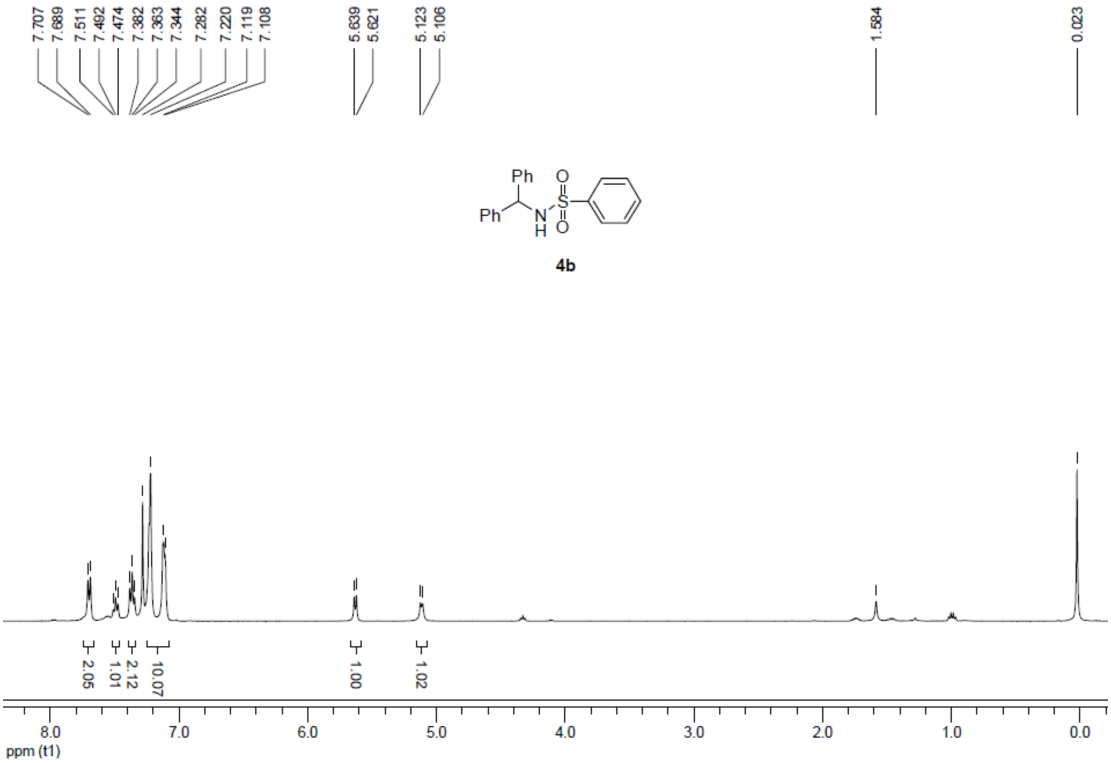
7.707
7.689
7.511
7.492
7.474
7.382
7.363
7.344
7.282
7.220
7.119
7.108
5.639
5.621
5.123
5.106
1.584
0.023
Ph
Ph
N
H
S
O
O
4b
2.05
1.01
2.12
10.07
1.00
1.02
8.0
7.0
6.0
5.0
4.0
3.0
2.0
1.0
0.0
ppm (t1)

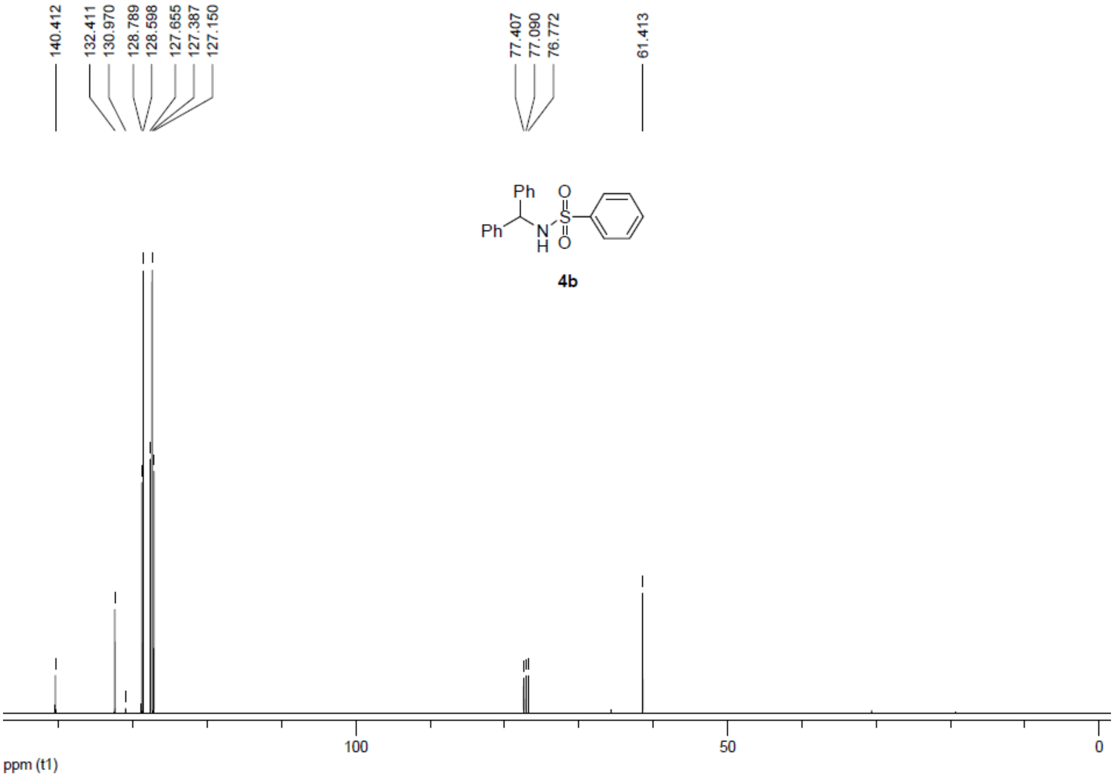
140.412
132.411
130.970
128.789
128.598
127.655
127.387
127.150
77.407
77.090
76.772
61.413
Ph
Ph
N
H
S
O
O
4b
100
50
0
ppm (t1)

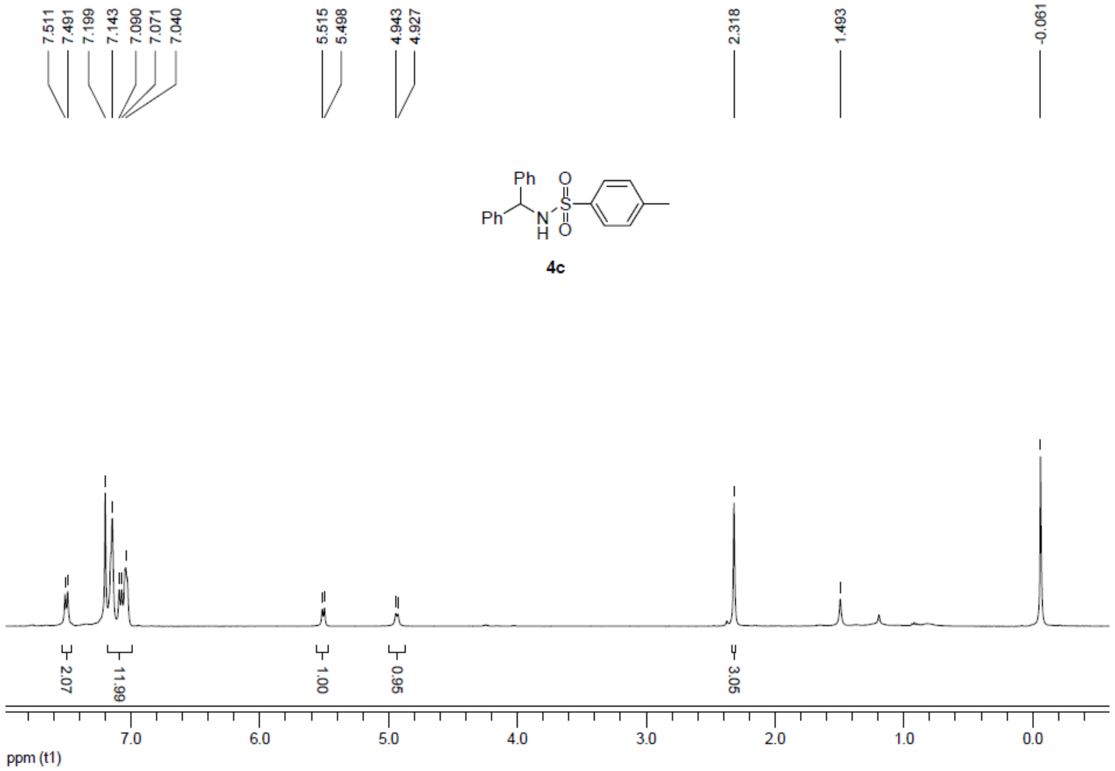
7.511
7.491
7.199
7.143
7.090
7.071
7.040
5.515
5.498
4.943
4.927
2.318
1.493
-0.061
Ph
Ph
N
H
S
O
O
4c
2.07
11.99
1.00
0.95
3.05
7.0
6.0
5.0
4.0
3.0
2.0
1.0
0.0
ppm (t1)

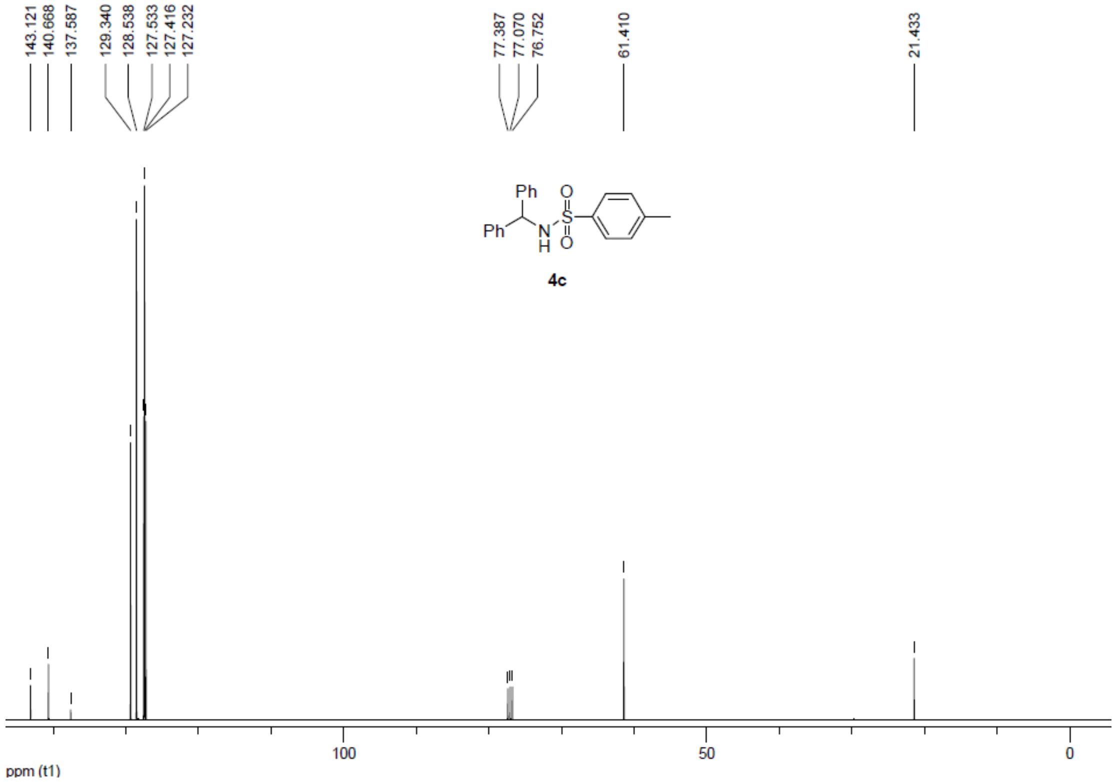
143.121
140.668
137.587
129.340
128.538
127.533
127.416
127.232
77.387
77.070
76.752
61.410
21.433
Ph
Ph
N
H
S
O
O
4c
100
50
0
ppm (t1)

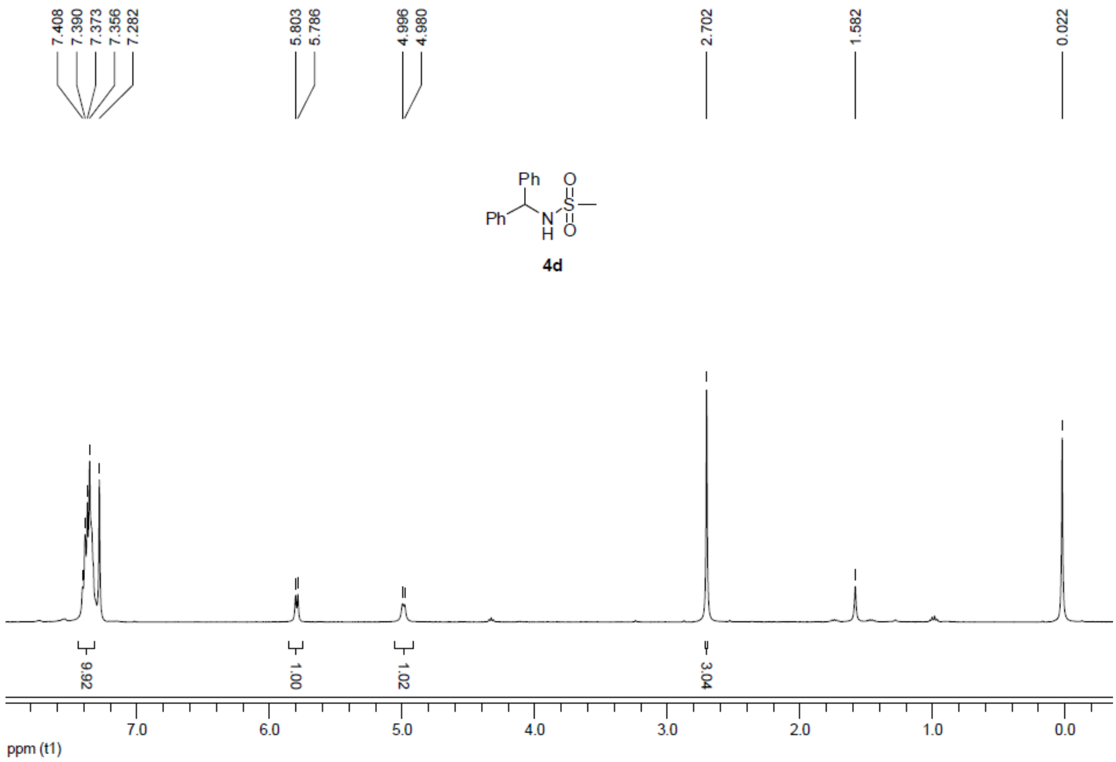
7.408
7.390
7.373
7.356
7.282
5.803
5.786
4.996
4.980
2.702
1.582
0.022
Ph
Ph
N
H
S
O
O
4d
9.92
1.00
1.02
3.04
7.0
6.0
5.0
4.0
3.0
2.0
1.0
0.0
ppm (t1)

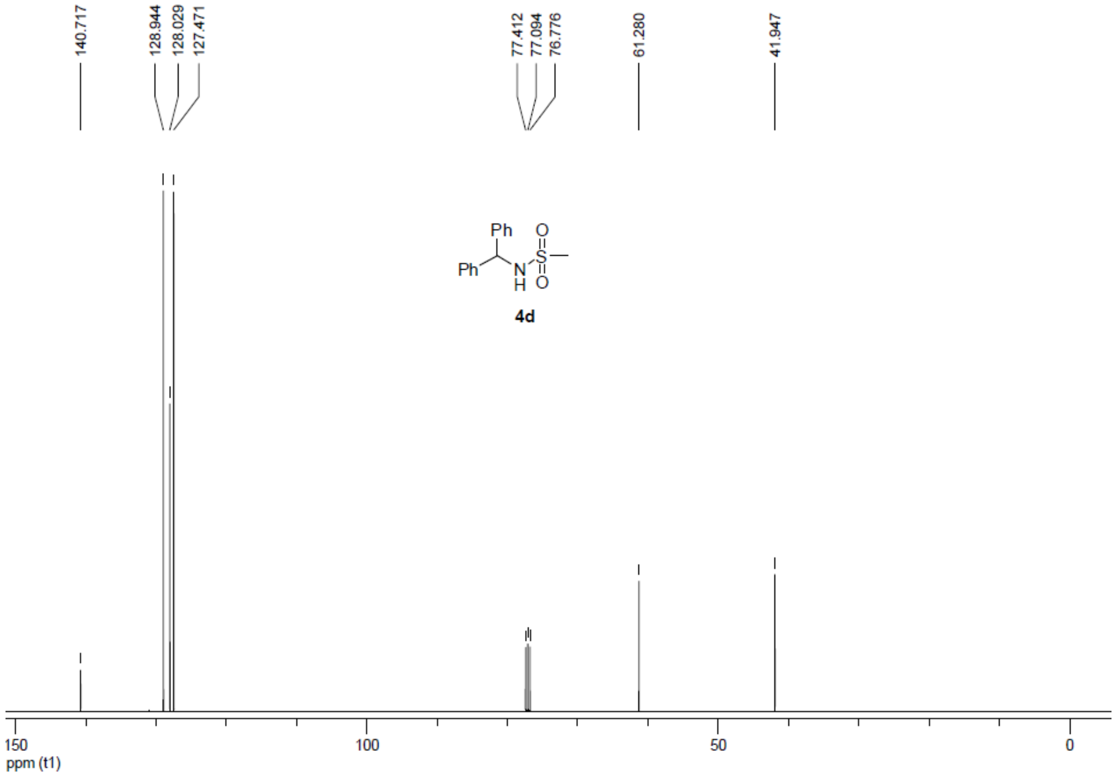
140.717
128.944
128.029
127.471
77.412
77.094
76.776
61.280
41.947
Ph
Ph
N
H
S
O
O
4d
150
100
50
0
ppm (t1)
